# Ultrasound and x-ray imageable poloxamer-based hydrogel for loco-regional therapy delivery in the liver

**DOI:** 10.1038/s41598-024-70992-5

**Published:** 2024-09-03

**Authors:** Jose F. Delgado, Joshua W. Owen, William F. Pritchard, Nicole A. Varble, Tania L. Lopez-Silva, Andrew S. Mikhail, Antonio Arrichiello, Trisha Ray, Robert Morhard, Tabea Borde, Laetitia Saccenti, Sheng Xu, Jocelyne Rivera, Joel P. Schneider, John W. Karanian, Bradford J. Wood

**Affiliations:** 1 Center for Interventional Oncology, Radiology and Imaging Sciences, Clinical Center, National Institutes of Health, Bethesda, MD USA; 2 Fischell Department of Bioengineering, University of Maryland, College Park. Maryland, USA; 3 Philips Healthcare, Cambridge, MA USA; 4 Chemical Biology Laboratory, Center for Cancer Research, National Cancer Institute, National Institutes of Health, Frederick, MD USA; 5 Department of Diagnostic and Interventional Radiology, UOS of Interventional Radiology, Ospedale Maggiore Di Lodi, Largo Donatori del Sangue, Lodi, Italy; 6 Institute of Biomedical Engineering, St. Catherine’s College, University of Oxford, Oxford, UK

**Keywords:** Cancer, Medical research, Oncology, Engineering

## Abstract

Intratumoral injections have the potential for enhanced cancer treatment efficacy while reducing costs and systemic exposure. However, intratumoral drug injections can result in substantial off-target leakage and are invisible under standard imaging modalities like ultrasound (US) and x-ray. A thermosensitive poloxamer-based gel for drug delivery was developed that is visible using x-ray imaging (computed tomography (CT), cone beam CT, fluoroscopy), as well as using US by means of integrating perfluorobutane-filled microbubbles (MBs). MBs content was optimized using tissue mimicking phantoms and ex vivo bovine livers. Gel formulations less than 1% MBs provided gel depositions that were clearly identifiable on US and distinguishable from tissue background and with minimal acoustic artifacts. The cross-sectional areas of gel depositions obtained with US and CT imaging were similar in studies using ex vivo bovine liver and *postmortem *in situ swine liver. The gel formulation enhanced multimodal image-guided navigation, enabling fusion of ultrasound and x-ray/CT imaging, which may enhance targeting, definition of spatial delivery, and overlap of tumor and gel. Although speculative, such a paradigm for intratumoral drug delivery might streamline clinical workflows, reduce radiation exposure by reliance on US, and boost the precision and accuracy of drug delivery targeting during procedures. Imageable gels may also provide enhanced temporal and spatial control of intratumoral conformal drug delivery.

## Introduction

Liver cancer is the 6th most common cancer worldwide^1^. Hepatocellular carcinoma, the predominant form of liver cancer, is treated at advanced stages with intravenously administered molecular targeted therapies and immunotherapy^2^; however, these treatments are limited by systemic side effects, low response rates, and high costs^3,4^. Intratumoral drug injections may mitigate systemic toxicities and can achieve high intratumoral drug concentrations, reducing total drug dose or systemic exposure compared to systemic administration^5,6^. However, this strategy faces a significant challenge due to the leakage of drug to adjacent untargeted tissues^5,7^. To address this challenge, hydrogels may be use to increase the viscosity of drug formulations, to better retain, localize, and deliver drugs by creating a semi-solid drug-eluting depot for improved control of intratumoral drug distribution^7–9^.

Hydrogels are increasingly used in cancer therapy for targeted delivery of anti-cancer agents, to try to enhance efficacy and reduce systemic side effects^10–12^. There are a variety of hydrogel materials that form a gel in response to biologic or external stimuli, enabling controlled drug release^13–21^. Techniques like intratumoral and transarterial delivery of drug-gel constructs have been previously explored^14,16,22–28^. Poloxamer 407 (POL), a temperature-responsive forming gel, transitions from liquid at room temperature to gel at body temperature, making it suitable for drug delivery^13,14,24,29^. The performance of POL, that incorporated iodinated contrast for x-ray-based imaging, using different needle devices and techniques was tested with a high level of morphological predictability after injections in ex vivo bovine liver^30^. Incorporating other contrast agents may improve gel and tissue visualization to enhance image-guided drug delivery procedures and to assess treatment zones.

Despite the effectiveness of x-ray imaging techniques like computed tomography (CT) for assessing drug delivery using imageable hydrogels^5^, ultrasound (US) imaging offers an alternative that is less expensive and permits real-time monitoring of gel delivery^31^. However, differentiating gels from surrounding tissues under US can be challenging due to the similar acoustic properties and impedance levels between tissue and the gel^32^. The incorporation of microbubbles (MBs) into POL gels aims to overcome this obstacle. These lipid-coated gas spheres resonate under US, enhancing the contrast between the gel and tissue, and thereby improving the visibility of volumes with gel delivery^33–38^.

MBs have been used in POL for visualization and inner ear drug delivery^39^. Nevertheless, the application of MBs within POL gels for delivery in deep organs has not been described. Herein, the development of MBs- and iodine-loaded POL gel for US and x-ray image-guided intratumoral delivery of drugs in the liver is reported. This work aims to develop a delivery vector that can increase the precision of drug delivery while minimizing radiation dose by means of enabling fusion of US and x-ray imaging. This innovation enables real-time visualization of the gel distribution within tumors via US imaging. Combining US and x-ray imaging with MBs and iodine-loaded POL gels, either used in parallel with image fusion or separately may facilitate more precise, image-guided drug delivery to tumors. This work supports further study of the effects upon treatment outcomes with the addition of gel, as well as the effects upon procedural times, radiation dose, and costs, given the imaging empowerment provided real-time US and multimodal image fusion and navigation systems^40–44^.

This study demonstrates the development of MBs- and iodine- loaded POL gel for US and x-ray image-guided localized delivery of drugs in the liver. This work aims to characterize the US and x-ray imageability of POL gel in vitro and in tissue for the precise drug delivery while also minimizing radiation dose by means of the fusion of US and x-ray/CT imaging.

## Results

### Development and characterization of POL containing microbubbles

POL formulation prepared as 22% (w/v in normal saline) transitions from liquid to gel at physiological temperature (37 °C). Spherical MBs, ranging from 2 to 20 µm (Fig. S1A), were successfully integrated into the POL gel. Gelation times varied with the percentage of MB concentration. The gelation time increased from 4 s ± 0.0 to 5.7 s ± 0.6 as the % MBs increased from 0 to 10%, All of the formulations gelled regardless of the % MB content (Fig. S1B). Microscopy confirmed MBs stability at both 21 °C and 37 °C (Fig. S1C-H).

**Fig. 1:**
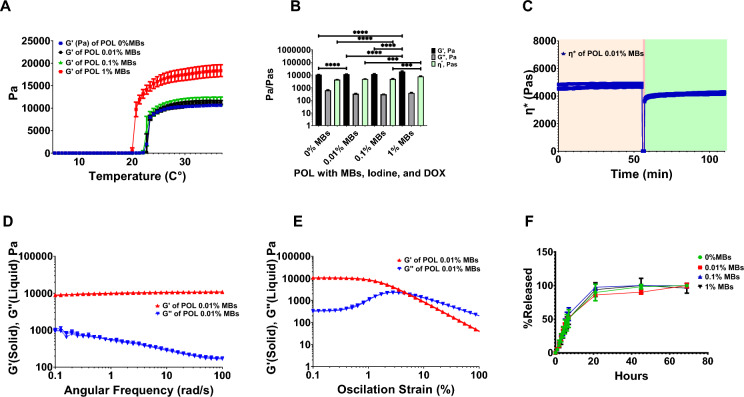
Characterization of rheological properties and DOX elution. (**A**) G’ (storage modulus) and G” (loss of modulus) during temperature ramp from 0 to 37 °C of POL gel with iodixanol (40 mg/mL of iodine), and DOX (10 mg/mL) containing 0%, 0.01%, 0.1%, and 1% MBs. (**B**) Viscoelastic properties of POL gel containing iodixanol and DOX: G’, G”, and ƞ* for 0%, 0.01%, 0.1%, and 1% MBs. (**C**) Thixotropic behavior of POL gel with iodixanol and DOX containing 0.01% MBs over time depicting ƞ* before strain (light tan), during strain (red), and after strain (green). (**D**) G’ and G” viscoelastic properties of POL with iodixanol and DOX containing 0.01% MBs over frequency domain. (**E**) G’ and G” viscoelastic properties of POL with iodixanol and DOX containing 0.01% MBs over oscillation strain sweep. (**F**) DOX elution for POL with 10 mg/mL DOX and iodixanol containing 0%, 0.01%, 0.1%, and 1% MBs. ***p < 0.001, ****p < 0.0001, from one-way ANOVA statistical test. Error bars represent means and standard deviations. n = 3 for all measurements.

Rheological assessment was performed to determine the gelation temperature of the POL containing iodine, doxorubicin (DOX), and varying concentrations of MBs, and to specifically assess its ability to gel at or below 37 °C (Table 1). All the tested formulations gelled below 37 °C (Fig. 1A). The addition of MBs to the POL gel containing 40 mg/mL of iodine from iodixanol^45^ and 10 mg/mL of DOX tended to decrease the gelation temperature with the greatest change shown with 1% (v/v) MB, the highest concentration tested. (Fig. 1A). The gelation temperatures were 23.0 ± 0.34 °C, 23.3 ± 0.06 °C, 22.7 ± 0.3 °C, and 20.8 ± 0.1 °C for 0%, 0.01%, 0.1%, and 1% (v/v) MBs, respectively. Significant differences in gelation temperatures were observed across formulations with varying percentages of MBs. Specifically, there were differences between 0% MBs and 0.01% MBs, 0% MBs and 1% MBs, 0.01% MBs and 1% MBs, as well as 0.1% MBs and 1% MBs, p < 0.0001 for all. However, there was no difference between 0.01 and 0.1% MBs (*p* = 0.52). The gelation temperatures changed depending on the presence or absence of MBs, or iodixanol (Fig.S2). G” across the formulations with DOX and containing MBs and iodixanol, remained stable and very similar after the gelation transition temperature regardless of the absence or presence of MBs (Fig.S3).

**Table 1:** Summary of POL characterization containing DOX, iodixanol, and varying MBs*.

Rheological measurement	% MBs (v/v)			
	0	0.01	0.1	1
Gelation temperature (C°)	23.0 ± 0.34	23.3 ± 0.06	22.7 ± 0.3	20.8 ± 0.1
G’ (Pa)	10,637.3 ± 214.8	11,653.6 ± 358.0	11,886.9 ± 910.7	18,835.8 ± 1748.0
tanδ	0.06 ± 0.00	0.06 ± 0.00	0.05 ± 0.00	0.04 ± 0.00
η* (Pa·s)	4350.0 ± 88.3	4759.5 ± 146.3	4854.4 ± 371.8	7691.3 ± 713.7
η* recovered after strain (%)	77.0 ± 3.4	76.8 ± 1.8	79.0 ± 1.4	73.1 ± 3.7
Flow point (%)	6.0 ± 0.8	4.4 ± 0	4.4 ± 0	6.1 ± 0.8
In vitro 50% DOX elution time (h)	7.8 ± 3.5h	6.5 ± 0.8 h	6.3 ± 1.8 h	7.0 ± 1.4

*Constant concentrations of 10 mg/mL DOX and 40 mg/mL of iodine from iodixanol.

The viscoelastic properties of the POL gel containing MBs, iodixanol, and DOX varied with the % MBs (Fig. 1B). Differences in storage moduli (G’) were found when comparing 0.01% and 0.1% to 1% MBs (*p* < 0.0001). G' values were 10,637.3 ± 214.8 Pa (0%), 11,653.6 ± 358.0 Pa (0.01%), 11,886.9 ± 910.7 Pa (0.1%), and 18,835.8 ± 1748.0 Pa (1% MBs). The tanδ values indicated the formation of hydrogels with a higher solid-like character (G’) than liquid-like properties (G’’) of POL at 37 °C across all MBs concentrations: 0.06 ± 0.00 (0%), 0.06 ± 0.00 (0.01%), 0.05 ± 0.00 (0.1%), and 0.04 ± 0.00 (1%). Complex viscosity (η*) increased with % MBs: 4350.0 ± 88.3 Pa·s (0%), 4759.5 ± 146.3 Pa·s (0.01%), 4854.4 ± 371.8 Pa·s (0.1%), and 7691.3 ± 713.7 Pa·s (1% MBs), with differences noted for 1% MBs compared to 0% (*p* < 0.0001), 0.01% (*p* = 0.0004 ) and 0.1% (*p* = 0.0005). All POL gel formulations with MBs, iodine, and DOX displayed viscoelastic characteristics (Fig.S4). The rheological characterization confirmed that all POL formulations with MBs formed hydrogels below physiological temperature and could be injected without affecting their mechanical properties.

POL gel containing iodixanol, DOX, and MBs exhibited thixotropic behavior (Fig. 1C) and were injectable even at body temperature (37 °C). POL gel containing 0.01% of MBs was able to flow under high shear stress and then reformed a gel. The ƞ* before strain at 37 °C was 7691.3 ± 713.7 Pa·s and immediately after strain was 4759.5 ± 146.3 Pa·s, recovering 76.8% ± 1.8of its ƞ*. All POL formulations without iodixanol (Fig.S5), with iodixanol (Fig.S6) and with iodixanol and DOX (10mg/mL) (Fig.S7) recovered their G’ after a high strain event independently of the addition of MBs, therefore, they are thixotropic. POL gel with iodixanol and DOX remained stable over the frequency domain as G’ was higher than G” (Fig. 1D) with a flow point of 4.4 ± 0% (Fig. 1E). The flow point of POL changed depending on the presence of iodixanol and/or DOX (Fig.S8). All the gels remained stable over the frequency domain as G’ was higher than G” (Fig.S9).

MBs concentration did not affect the release of DOX at 10 mg/mL in the presence of iodixanol (Fig. 1F). Fifty percent of DOX eluted at 7.8 ± 3.5h, 6.5 ± 0.8 h, 6.3 ± 1.8 h, and 7.0 ± 1.4 h for 0%, 0.01%, 0.1%, and 1% MBs (v/v), showing no differences (*p* = 0.9). In general, POL with DOX (10 mg/mL) showed a sustained release pattern across all MB concentrations tested.

Additional information pertaining to rheological studies can be found in Table S1 and S2).

### US imaging assessment with tissue mimicking phantoms

Percent MBs in POL gel was optimized using tissue mimicking phantoms (TMP) (Fig.S10). Imaging 1% MBs produced images with acoustic intensities similar to that for 5%, and 10% MBs in POL gel and were clearly distinguished from background. In addition, the acoustic heterogeneities and entropies were similar to 5%, and 10% MBs in POL. Acoustic heterogeneity measured how much the pixel intensities deviated from the mean pixel intensity in the selected region of interest (ROI). Entropy is the measure of randomness in the position of pixel values in an ROI (Fig. S10). POL with 1% MBs also provided areas with decreased acoustic artifacts such as reflective gray areas deep to the POL location (comet tails). Due to these results, POL gel with % MBs below 1% was further tested ex vivo.

### Vascular occlusion and dislodging of gel

In the event of POL gel leaking into blood vessels, an agar-based vascular phantom was occluded with POL gel with varying concentration of MBs and the pressure required to dislodge the gel with normal saline was measured. There was a tendency toward a decrease in the required pressure with increasing % MBs (p > 0.09) (Fig. S11).

### Assessment of US imageability of POL in ex vivo tissue

POL gels with 0.001, 0.01, and 0.1% MBs, were imaged after injection in ex vivo bovine liver. This range was selected for study as these lower % MBs provided a clear delineation of gel from surrounding tissue on US without introducing significant acoustic artifacts (Fig. S12). The entropy of the images of the POL gels, a statistical measure that can be used to characterize the texture of the input image, was lower in POL with 0.01% MBs compared to 0.001% MBs (*p* = 0.0389) (Fig. S12F). From this optimization, 0.01% MBs formulation was selected for further testing (Fig. S12).

**Fig.2:**
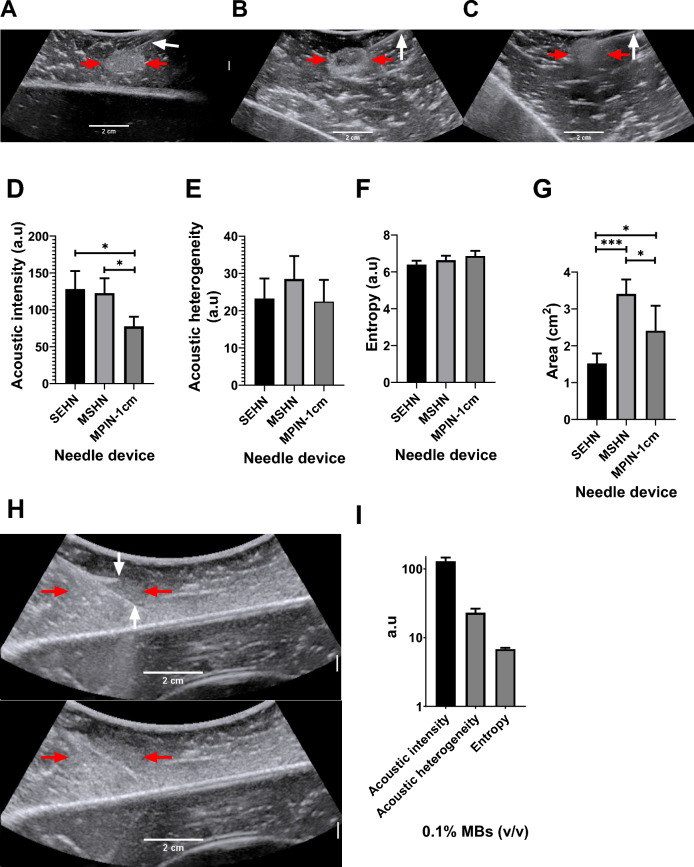
US imaging of 4 mL POL containing MBs injected into bovine liver ex vivo*.* US image after injection of POL containing 0.01% MBs at 100 mL/hr using (**A**) SEHN, (**B**) MSHN, and (**C**) MPIN -1 cm showing the POL deposition (red arrow) and the needle shaft (white arrow). For the MPIN-1 cm, the based delivery needle is indicated. (**D**) Acoustic intensities, (**E**) heterogeneities, (**F**) entropy, and (**G**) area of injected POL following injection with different needle devices. (**H**) MPIN-1 cm injection, 0.1% MBs following POL injection (4 mL, 100 mL/h). The top panel partially shows a deployed needle and the central shaft (white arrows). The margins of the gel are very ill-defined (red arrows). The lower panel shows the gel after removal of the needles. (**I**) Acoustic intensity, heterogeneity, and entropy of (G). *p < 0.05, ***p < 0.001 from one-way ANOVA statistical test. Error bars represent standard deviations of average (n = 5 for SEHN, n = 4 for MSHN, and n = 3 for MPIN-1cm).

The US imageability of the POL gel depot in the bovine liver depended on the needle device used for injection (Fig. 2). All tested injections resulted in localized depot formation with clear delineation and hyperechogenicity on US. POL gel containing 0.01% MBs was injected (4 mL, 100 mL/h) into ex vivo bovine tissue using a single end hole needle (SEHN), multiple side hole needle (MSHN), and multiprong injection needle (MPIN) deployed 1 cm (MPIN-1 cm) (Fig. 2A–C). For MPIN-1 cm, the US pixel intensity was lost by 32.7 ± 12.9% and 40.4 ± 14.3% compared to SEHN (*p* = 0.0233) and MSHN (p = 0.0495), respectively. The acoustic intensities, representing pixel intensities, were 128.1 ± 24.6, 122.6 ± 20.2, and 77.7 ± 13.0 a.u (arbitrary unit) for POL gel injected with SEHN, MSHN, and MPIN (Fig. 2D). The acoustic heterogeneity in tissue of POL gel across the tested needle devices was similar (*p* > 0.4) (Fig. 2E). The acoustic heterogeneities were 23.3 ± 4.8, 28.5 ± 5.3, and 22.4 ± 4.8 a.u (arbitrary unit) for POL gel injected with SEHN, MSHN, and MPIN-1 cm, respectively. Due to the loss of US imageability after injection with MPIN, the amount of MBs was increased by tenfold to 0.1% MBs (Fig. 2H) resulting in an acoustic intensity of 129.5 ± 16.6 a.u, an increase of 1.7 times compared to with 0.01% MBs (*p* = 0.0068) (Fig. 2I). However, the acoustic heterogeneity of POL gel containing 0.1% MBs, remained similar (23.1 ± 3.3 a.u) to POL gel containing 0.01% MBs (Fig. 2I). The US image entropy of 0.01% MBs-containing POL across the three needle devices was not statistically significant (*p* = 0.44) (Fig. 2F). The treatment cross-sectional areas calculated based on ultrasound imaging were 1.5 ± 0.3, 3.4 ± 0.4, and 2.4 ± 0.7 cm^2^ for POL gel injected with SEHN, MSHN, and MPIN-1 cm, respectively (Fig. 2G). These results were different when comparing the needles to each other: SEHN vs MSHN, p = 0.0003; SEHN vs MPIN-1 cm, *p* = 0.0478, and MSHN vs MPIN-1 cm, p = 0.0342. The entropy of 0.1% MBs, also remained similar with 6.8 ± 0.3 a.u compared to POL gel with 0.01% MBs with 6.9 ± 0.3 a.u (Fig. 2I).

### Spatiotemporal US analysis of POL gel in ex vivo tissue

**Fig.3:**
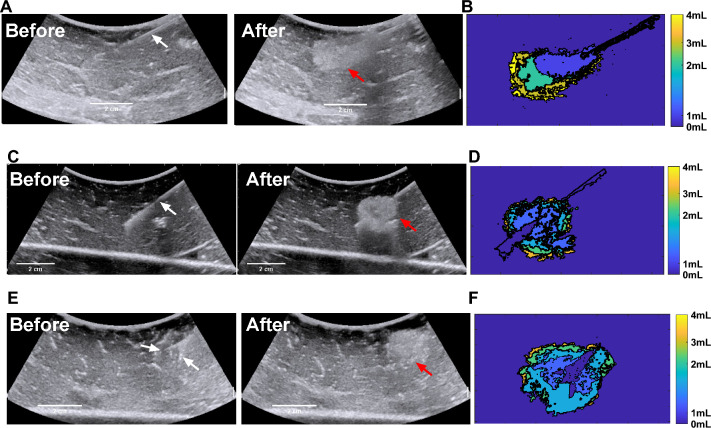
Representative spatiotemporal US images during injection of 4 mL POL containing 0.01% v/v MB at 100 mL/hr. US before injection with the needle (white arrows) in place, gel after injection (red arrows) with the needle removed, and rendering of incremental areas for cumulative injected volumes at 1, 2, 3, and 4 mL for SEHN (**A** and **B**), MSHN (**C** and **D**) and MPIN -1 cm (**E** and **F**). MPIN-1 cm US (**E**) images before injection show the main needle and a deployed prong.(white arrows), and injected volume (red arrow). .

**Fig.4:**
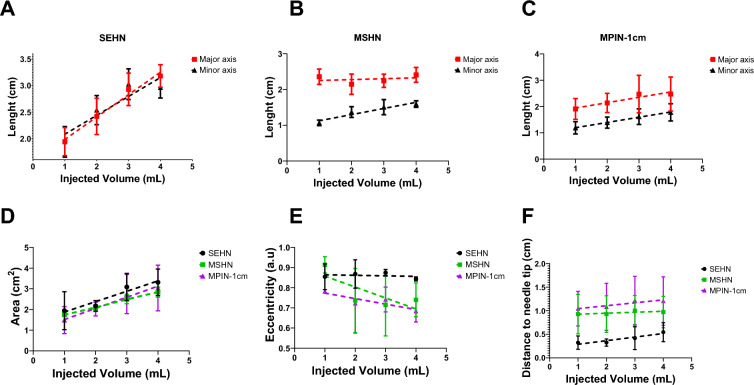
Geometric changes during injection of 4 mL of POL, measured at 1 mL increments of injected volume. Lengths of major and minor axes of POL deposited in tissue per 1 mL increment injected volume with SEHN (**A**), MSHN (**B**), and MPIN-1 cm (**C**). (**D**) Cross-sectional area of POL deposited in tissue per mL injected with SEHN, MSHN, and MPIN-1 cm. (**E**) Eccentricities of POL deposited in tissue per mL injected with SEHN, MSHN, and MPIN-1 cm. (**F**) Distance from the centroid of POL deposited in tissue to the needle tip per each 1 mL with SEHN, MSHN, and MPIN-1 cm. For the case of MPIN-1 cm, the distance was measured in respect to the major needle tip. All 4 mL injections were performed at 100 mL/h. Error bars represent standard deviations for n = 3 average. Dotted lines represent linear regressions. Multivariable linear regression was used to calculate regression coefficients (n = 3) for (**F**). Correlation matrices were obtained by computing Pearson correlation coefficients (Average of n = 3) for (**F**).

The dimensions of POL gel injected into ex vivo tissue as measured by US imaging were proportional to the injected volume (Figs. 3 and 4). The lengths of the minor and major axes for POL depots for the three needle devices are shown (Fig. 4A–C). The cross-sectional areas of the injected POL gel were calculated after 1 mL increments of injected volume to a total of 4 mL. The areas for the three needles were 1.9 ± 0.9, 2.2 ± 0.3, 3.1 ± 0.6, and 3.3 ± 0.6 cm2 with SEHN; 1.7 ± 0.0, 2.1 ± 0.1, 2.5 ± 0.2, and 2.8 ± 0.2 cm2 for MSHN; and 1.5 ± 0.6, 2.0 ± 0.3, 2.7 ± 0.9, and 3.0 ± 1.1 cm2 for MPIN-1 cm for the cumulative 1, 2, 3, and 4 mL injections, respectively (Fig. 4D). There were linearly increasing relationships between area and injected volume for the three needles: y = 0.5x + 1.37 (R2 = 0.9) for SEHN, y = 0.4x + 1.4 (R2 = 1.0) for MSHN, and y = 0.5x + 1.0 (R2 = 1.0) for MPIN-1 cm. Eccentricity of POL gel injections was consistent across needle devices per mL injected (Fig. 4E). For SEHN, eccentricities ranged from 0.8 ± 0.1 a.u to 0.8 ± 0.0 a.u for 1 and 4 mL. MSHN showed 0.9 ± 0.0 to 0.7 ± 0.1, and MPIN-1 cm showed 0.8 ± 0.0 a.u to 0.7 ± 0.0 a.u across the same volumes.

Distance from the POL gel deposition center to the needle tip also remained tended to increase with injection volume (Fig. 4F)., with SEHN distances from 0.3 ± 0.1 to 0.5 ± 0.2 cm, MSHN from 0.9 ± 0.4 to 1.0 ± 0.3 cm, and MPIN-1 cm from 1.0 ± 0.4 to 1.2 ± 0.5 cm for 1 to 4 mL injections, respectively. The relative effects in the distance of the center of the POL injection to the needle tip in the volume suggested to be different depending on the needle device used. The weighted effect of SEHN in the distance to needle tip suggested to be higher compared to the other needle devices (See regression coefficients in Table S3), nevertheless, no differences were obtained (*p* > 0.3) (Table S4). In addition, the distance of POL center to the needle tip values per mL injected correlated across all the needle devices (Fig. S13). Additional information of statistical analysis pertaining to the distance of needle tip to POL injection center can be found in Table S5 and S6.

The solidities (concavity degree) for the areas analyzed per mL of POL gel are available in Fig. S14.

### Injection techniques monitored under US in ex vivo tissue

**Fig.5:**
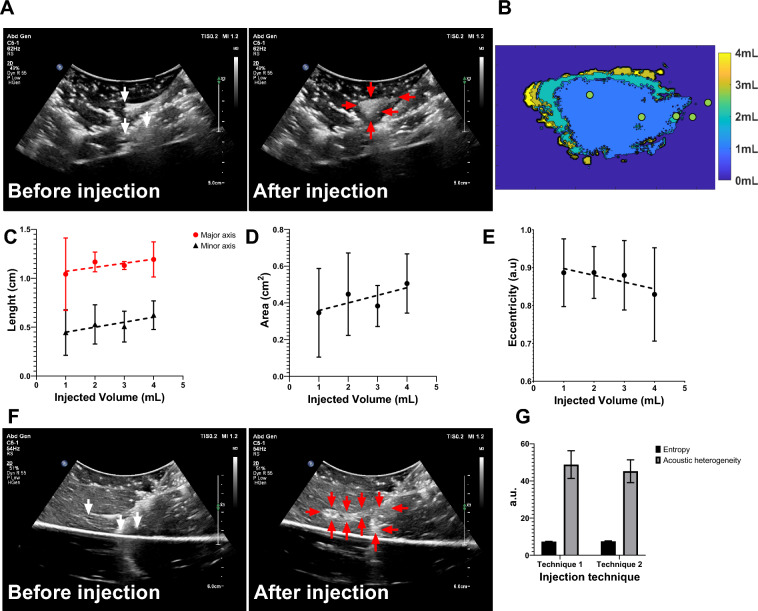
Spatiotemporal distribution of two MPIN injection techniques before and after POL gel injection for POL deposited from the selection of one of the three prongs. (**A**) US images in B-mode before injection with the MPIN in place with the three needles deployed 2 cm (white arrows points needle tips) and then after completion of the injection of POL while the needles are retracted from 2 to 1 cm tip to tip and removal of the needle (red arrows points gel deposition). (**B**) Color-coded POL deposition progression per 1 mL increment of the injected volume shown in (**A**). Green dots of (**B**) depicts needle tip retraction track. For the injection in (**A**), the 1 mL incremental values for (**C**) lengths of major and minor axes, (**D**) cross-sectional area, and (**E**) eccentricities are shown. (**F**) US images in B-mode of MPIN needles before injection (red arrows points gel) and then after needle retraction from 5 to 1 cm while injecting POL gel and needle removal (red arrows points gel deposition). (**G**) Entropy and acoustic heterogeneity values of (**A**), and (**F**) at final 4 mL POL injection. Error bars represent standard deviations for n = 3 average. Dotted lines represent linear regressions.

Two injection techniques with MPIN were imageable under US and POL deposition in tissue was evaluated for different needle retraction positions (See injection techniques in methods section) (Fig. 5). For the first injection technique (Fig. 5A), the calculated areas of the POL depositions (Fig. 5B) of each of the three needles during retraction were 0.3 ± 0.2, 0.4 ± 0.2, 0.4 ± 0.1, and 0.5 ± 0.2 cm^2^ for 1, 2, 3, and 4 mL respectively, where the results of one of the three individual needles were reported (Fig. 5C). The major axis lengths of deposited POL were 1.0 ± 0.3, 1.2 ± 0.1,1.1 ± 0.04,1.2 ± 0.2 cm for 1, 2, 3, and 4 mL respectively (Fig. 5D). The minor axis lengths were 0.4 ± 0.2, 0.5 ± 0.2, 0.5 ± 0.1, 0.6 ± 0.1 cm for 1,2,3, and 4 mL respectively (Fig. 5D). The eccentricity for all incremental injections was 0.9 ± 0.1 for 1,2, and 3 mL and 0.8 ± 0.1 for 4 mL (Fig. 5E).

For the second injection technique, (Fig. 5F), while monitoring the POL deposition, the US image was variably hypoechoic (dark regions) and hyperechoic. The track of injected POL was visible after the needle was removed due to elimination of needle artifacts. The POL deposition was in form of string of speckles with a 0.7 ± 0.1 cm major axis length and 0.5 ± 0.0 minor axis length.

The acoustic heterogeneity was calculated and was 48.8 ± 7.4 a.u and 45.2 ± 6.1 a.u. for the first and second technique, respectively (Fig. 5G). The entropies for both techniques were 7.3 ± 0.1 a.u., and 7.5 ± 0.2 a.u. for the first and second technique respectively (Fig. 5G).

### Transducer type evaluation of POL gel injected in ex vivo liver

**Fig.6:**
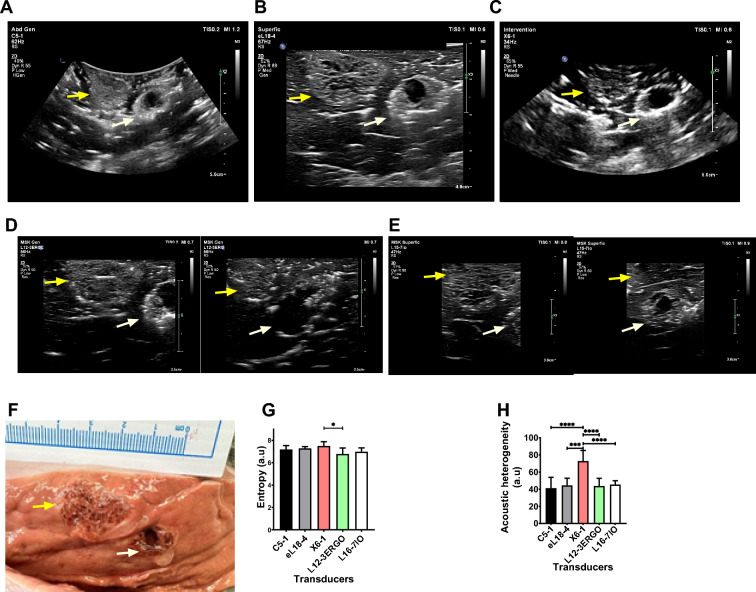
Evaluation of US imageability using 5 transducers. B-mode image of two adjacent 4 mL POL injections (yellow and white arrows) using (**A**) C5-1, (**B**) El18-4, and (**C**) X6-1 transducers. B-mode US images of the same adjacent injections with L12-3ERGO transducer (**D**, right, and left) and L16-7IO (**E**, right, and left). (**F**) Gross pathology of two 4 mL injected POL. (**G**) Entropy of the B-mode US images for 5 transducers and (H) corresponding acoustic heterogeneities. *p < 0.05, ***p < 0.001, ****p < 0.0001 from one-way ANOVA statistical test. Error bars represent standard deviations for n = 3 average.

Transducer evaluation for POL gel in ex vivo bovine liver showed that imaging detail varies by transducer types designed to work with different frequencies (Fig. 6). Two adjacent 4 mL POL injections were analyzed using five transducers (Philips Epiq): C5-1 (62 Hz), eL18-4 (67 Hz), X6-1 (34 Hz), L12-3ERGO (56 Hz), and L15-7io (50 Hz). The distance between the centroids of the two injections was of 2.2 ± 0.1 cm (Fig. 6A–E). Entropy was consistent across transducers except between X6-1 and L12-3ERGO (p = 0.0218) (Fig. 6G). X6-1 3D matrix displayed higher acoustic heterogeneity (73.0 ± 12.3 a.u.) than C5-1 curved, eL18-4 linear, L12-3ERGO linear, and L15-7io linear, (*p* values < 0.0001, 0.0002, 0.0002, and 0.0005, respectively) (Fig. 6H). Linear array transducers provided clearer details of gel over curvilinear ones, confirmed by gross pathology showing hypoechoic cavities and hyperechoic gel areas (Fig. 6F). eL18-4 and L15-7io transducers offered the highest detail. Optical microscopy of a POL-filled cavity confirmed its transition from solid to liquid, evidenced by an empty cavity in tissue (Fig. S15). The Fig. S16 also evidenced a cavity filled with POL and surrounded by tissue.

### Assessment of US imageability of POL gel containing DOX in ex vivo tissue

**Fig.7:**
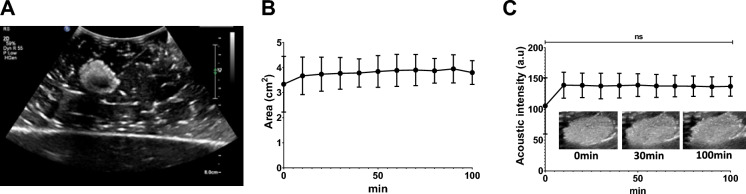
US imageability of POL with DOX, as well as imaging stability over time in ex vivo tissue. (**A**) B-mode US images of POL with 10 mg/mL of DOX. (**B**) Area of POL with 10 mg/mL of DOX over time. (**C**) Acoustic intensity from 0 to 100 min (**C**). Error bars represent standard deviations for n = 3 average. ns p > 0.999.

POL gel loaded with DOX 10 mg/mL was imageable under US (Fig. 7A) and was visible over 100 min (Fig. 7). The area of POL based on US imaging did not differ between 0 and 100 min after injection (*p* > 0.9999) (Fig. 7). The areas were 3.3 ± 0.9 cm^2^ and 3.8 ± 4 cm^2^ at 0 and 100 min, respectively (Fig. 7B). The acoustic intensity, similarly, was not different at the tested times (105.6 ± 37.2 a.u. at 0 min, and 137.3 ± 13.0 a.u. at 100 min) (*p* > 0.9999) (Fig. 7C).

### Assessment of US and x-ray imageability of POL gel in ex vivo tissue

**Fig.8:**
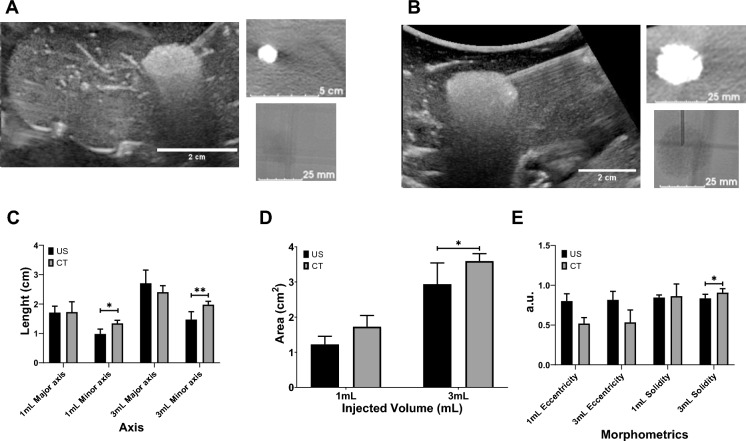
US and x-ray imageability of POL. (A) 1 mL of POL injected in ex vivo liver with US (left), CT (upper right), and fluoroscopic (lower right) images. (**B**) 3 mL of POL injected in ex vivo liver with US (left), CT (upper right), and fluoroscopic (lower right) images. (**C**) Length (cm) of major (equatorial) and minor (meridian) axes of 1 mL and 3 mL injected POL in ex vivo liver based on US and CT images. (**D**) Area (cm^2^) of 1 mL and 3 mL injected POL in ex vivo liver from US and CT images. (**E**) Eccentricity (a.u.) of 1 mL and 3 mL injected POL in ex vivo tissue from US and CT images. *p < 0.05, **p < 0.01 from t-test statistical analysis. Error bars represent standard deviations for n = 3 average.

POL gel was imageable under US and x-ray-based imaging, both CT and fluoroscopy. Both US and CT imaging showed similar morphology (Fig. 8A and B) and 2D measurements (Fig. 8C–E). Eccentricities were 0.8 ± 0.1, and 0.6 ± 0.2 for 1 mL POL measured from US and CT, respectively (Fig. 8E). For the 3 mL POL injections, the eccentricity values were 0.8 ± 0.1 a.u., and 0.5 ± 0.1 a.u. for US and CT, respectively. The solidities were 0.8 ± 0.0 a.u., and 0.9 ± 0.1 a.u. for 1 mL of POL injected, based on US and CT imaging, respectively (Fig. 8E). Finally, the solidities for 3 mL of POL injected were 0.8 + 0.0 a.u. and 0.9 ± 0.0 a.u. based on US and CT imaging, respectively (Fig. 8E).

**Fig.9:**
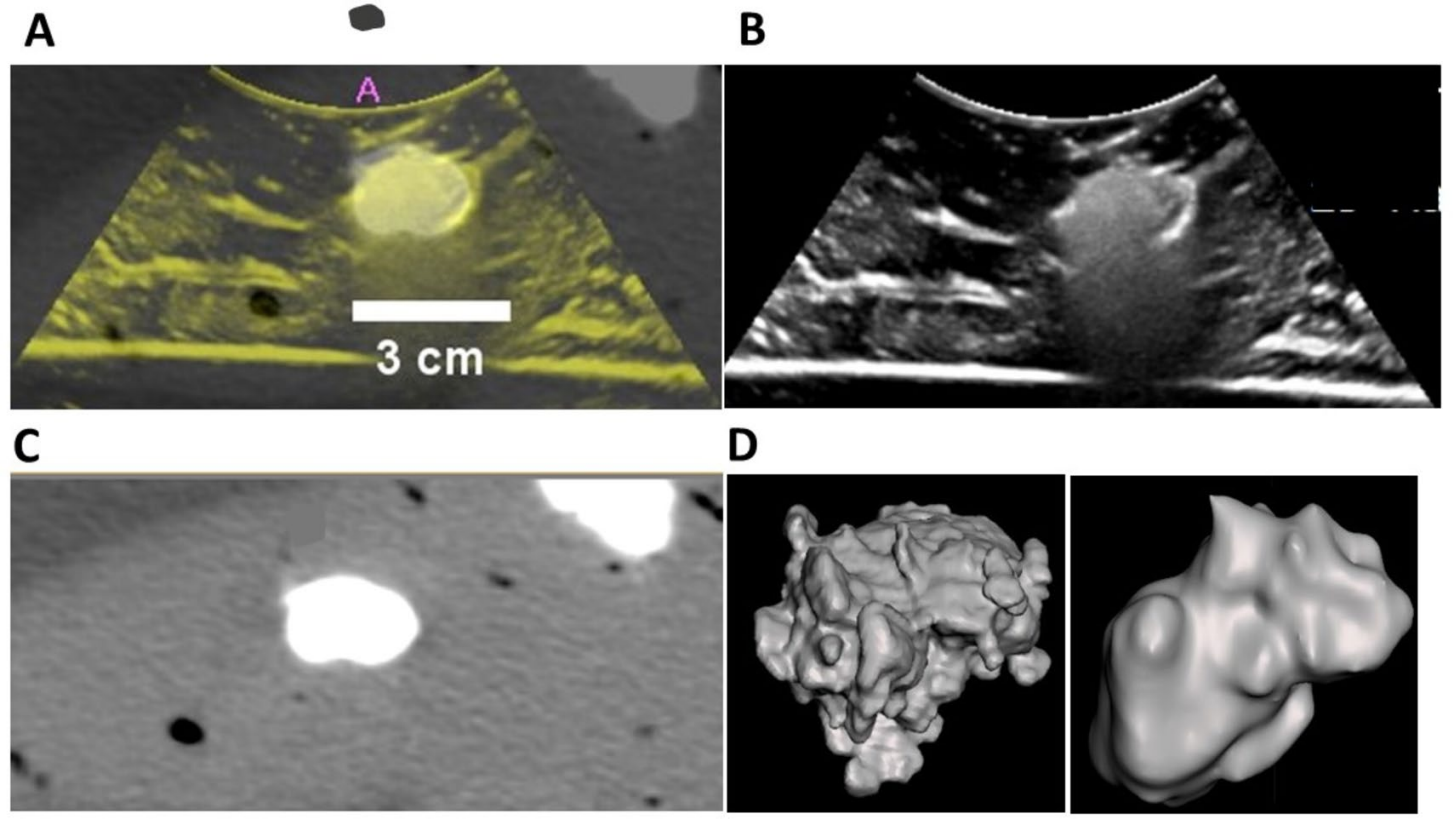
US and CT image fusion, and 3D distribution of POL from US and CT imaging. (**A**) US and CT co-registration from POL with 10 mg/mL (4 mL) injected with MSHN, and their individual US (**B**), and CT (**C**) images. (**D**) 3D distribution of POL gel obtained from US (left), and CT (right) imaging.

US and CT images of POL co-registered (dual imaging fusion overlapping) injected into ex vivo liver demonstrates dual imageability of POL (Fig. 9A–C). Qualitatively, both co-registered images matched the localization of POL. Linear structures, presumed to be vessels or fascial planes filled with air, were visualized under US imaging, but were not readily visible under CT.

3D distribution of POL from US sweeps with C5-1 transducer were similar to the shape derived from CT. Figure 9D depicts a representative 3D distribution of POL with 0.01% MBs injected with SEHN. The 3D images from US provided irregular and rugose surfaces compared to the smoother surfaces from CT.

**Fig.10:**
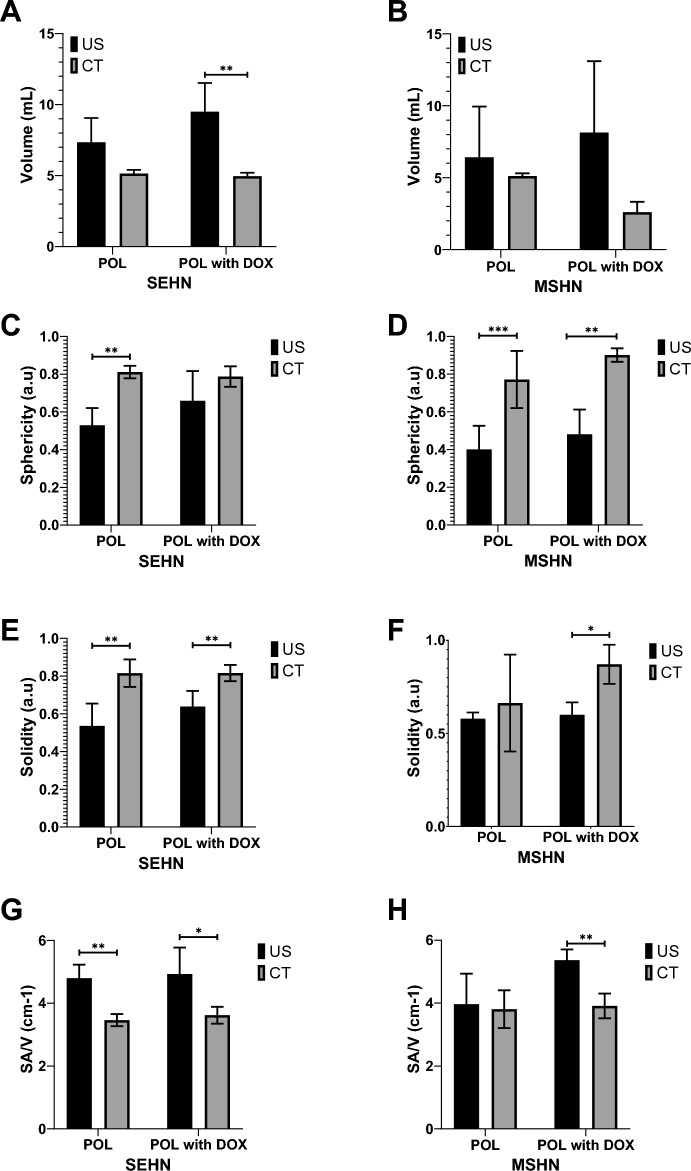
Volumetric, morphometric, and surface area-to-volume ratio of POL with and without DOX and comparison between US and CT imaging. Volume of POL with and without 10 mg/mL of DOX obtained from US and CT imaging for (**A**) SEHN, and (**B**) MSHN. Sphericity (**C**), solidity (**E**), and SA/V (**G**) are included for SEHN. Sphericity (**D**), solidity (**F**), and SA/V (**F**) are also included for MSHN. *p < 0.05, **p < 0.01, ***p < 0.001 from t-test statistical analysis.

Volume, sphericity, solidity, and surface-area-to-volume (SA/V) of POL gel with and without DOX revealed slight differences when comparing with US and CT 3D distributions (Table 2, I Fig. 10). Overall, the volumes of POL injections obtained with CT imaging (Fig. 10A and B), were lower than those of US imaging.

**Table 2:** Volume, sphericity, solidity, and SA/V parameters for POL gel with and without DOX.

Parameter	SEHN with DOX		SEHN without DOX		MSHN with DOX		MSHN without DOX	
	US	CT	US	CT	US	CT	US	CT
Volume	7.3 ± 1.7 mL	5.1 ± 0.3 mL	9.5 ± 2.0 mL	5.0 ± 0.2 mL	6.4 ± 3.5 mL	5.1 ± 0.2 mL	8.1 ± 5.0 mL	2.6 ± 0.7 mL
Sphericity	0.6 ± 0.1 a.u	0.8 ± 0.0 a.u	0.5 ± 0.1 a.u	0.8 ± 0.0 a.u	0.5 ± 0.1 a.u	0.9 ± 0.0 a.u	0.4 ± 0.1 a.u	0.8 ± 0.1 a.u
Solidity	0.5 ± 0.1 a.u	0.8 ± 0.1 a.u	0.6 + 0.1 a.u	0.8 + 0.0 a.u	0.5 ± 0.1 a.u	0.8 ± 0.1 a.u	0.6 ± 0.0 a.u	0.9 ± 0.1 a.u
SA/V	4.8 ± 0.4 cm^-1^	3.4 ± 0.2 cm^-1^	4.9 ± 0.8 cm^-1^	3.6 ± 0.3 cm^-1^	5.4 ± 0.3 cm^-1^	3.9 ± 0.4 cm^-1^	4.0 ± 1.0 cm^-1^	3.8 ± 0.6 cm^-1^

SA/V = surface area/volume ratio. Values are mean ± standard deviations (n = 3).

The sphericities of POL obtained from CT imaging were generally higher compared to US imaging (Fig. 10C and D). In addition, solidities of POL 3D distributions were higher from CT imaging compared to US imaging (Fig. 10E and F), but that could perhaps be due to volume averaging and lack of detection of small vessel incursion on CT. Finally, the SA/V tended to be higher for 3D distribution of POL injections obtained with US imaging compared to CT (Fig. 10G and H). However, the differences between results with US and CT could be due to the irregularity of the rendered surface on US. Many factors might contribute to the smoothness of the edge, including surface reconstructions, volume averaging, and smoothing algorithms.

There were some differences on volume, sphericity, solidity, and SA/V parameters in the majority of the formulations of POL with or without DOX after injection with SEHN or MSHN (Fig. 10).

### Assessment of US and x-ray imageability of POL in *postmortem* swine liver

**Fig.11:**
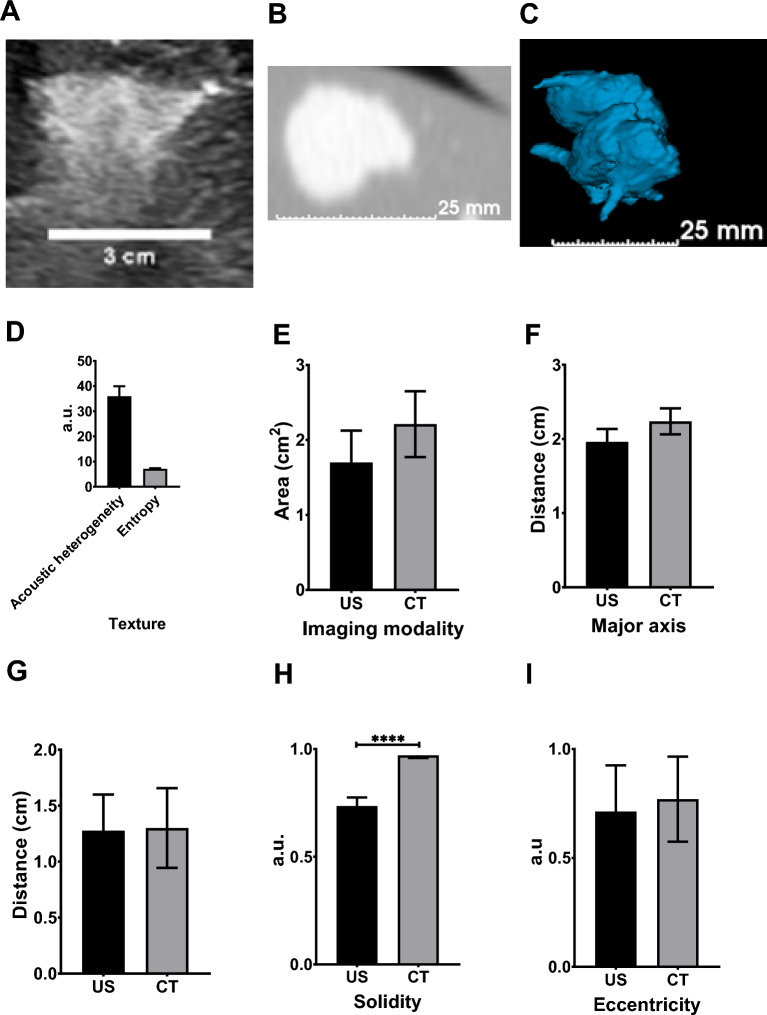
US and CT analysis of 3 mL of POL containing 10 mg/mL in *postmortem* tissue. (**A**) B-mode US image of POL injected, as well as its CT (**B**), and 3D distribution from CT (**C**). (**D**) Texture of POL images from US with their area I, major axis (**F**), minor axis (**G**), solidity (**H**), and eccentricity (**I**) from US and CT imaging. ****p < 0.0001 from t-test statistical analysis. Error bars represent standard deviations for n = 3 average.

As a prelude to in vivo evaluation of CT and US imageable POL in swine, the 2D distribution of POL with 10 mg/mL DOX in *postmortem* swine liver was tested and showed correlation between US and CT imaging (Fig. 11A and B). Four 3 mL SEHN injections were imageable and exhibited slight intravasation (Fig. 11C). Acoustic intensity was 144.03 ± 19.5 a.u., with acoustic heterogeneity at 36.0 ± 4.0 a.u. and entropy at 7.1 ± 0.2 a.u. (Fig. 11D). Areas were similar across modalities: 1.7 ± 0.4cm2 for US and 2.2 ± 0.4cm2 for CT (p = 0.1835) (Fig. 11E). Major axis lengths were 2.0 ± 0.2 cm for US and 2.2 ± 0.2 cm for CT (p = 0.0932) (Fig. 11F), with minor axis lengths at 1.3 ± 0.2 cm and 1.3 ± 0.3 cm, respectively (*p* = 0.9339) (Fig. 11G). Solidities were 0.7 ± 0.0 for US and 0.8 ± 0.0 for CT (*p* < 0.0001) (Fig. 11H), and eccentricities were 0.7 ± 0.2 for US and 0.8 ± 0.2 for CT (p = 0.7281) (Fig. 11I).

**Fig.12:**
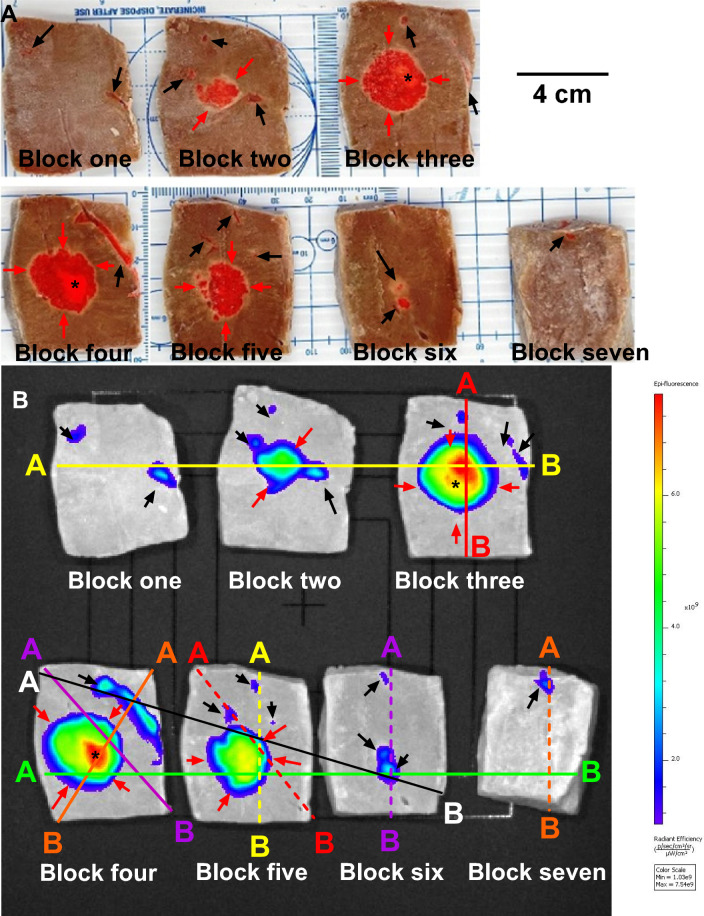

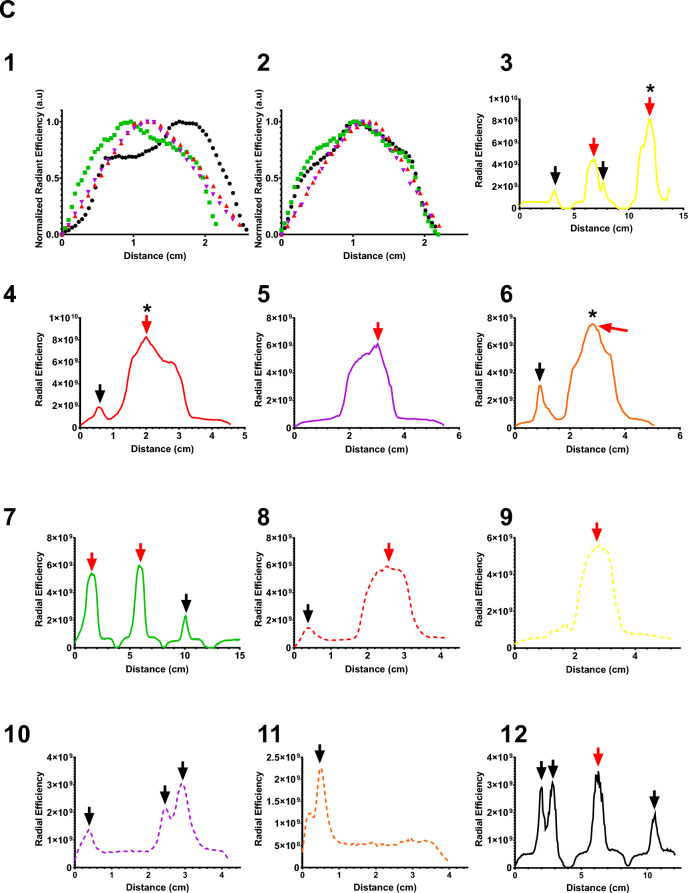
Gross Pathology and DOX Distribution of POL with DOX Injected into Tissue with MSHN. (**A**) Photograph of bovine liver tissue injected with POL containing DOX (2mg/mL) (n = 1) and cut into seven blocks. Black arrows indicate intravasation of POL with DOX, while red arrows denote localized deposition of POL with DOX. (**B**) Optical imaging of DOX fluorescence for the seven tissue blocks depicted in (A). Lines drawn correspond to ten regions colored differently, showing DOX radiant efficiency ([p/s/cm^2^/sr]/[µW/cm^2^]) over distance within the tissue blocks from (**A**) and (**B**). (**C**) Optical imaging showing DOX distribution along the short (1) and long axes (2) following POL containing DOX (2mg/mL) for localized injections with MSHN (n = 4). Sections (3–12) display optical imaging distribution over distance for the ten regions outlined in (**B**), represented by colored lines, for a representative POL injection containing DOX in bovine tissue (n = 1, selected from four injections performed with MSHN). Legend: Black arrows: Intravasation of POL with DOX; Red arrows: Localized deposition of POL with DOX, and (*) Depicts injection core.

A representative example of DOX-containing POL (Fig. 12 A and B) illustrates the localized deposition of POL and leaked material. Gross pathology of bovine liver samples revealed DOX contained within POL, forming tissue cavities at the injection core, with gel distributed in a circular shape (Fig. 12A). Intravasated gel was detected in vessels surrounding the injection site. Optical imaging of corresponding tissue blocks confirmed DOX distribution throughout tissue sections, with the majority of fluorescent DOX concentrated at the injection site and to a lesser degree in intravasated material. Figure 12 C1 and C2 depict the DOX distribution profiles across the long and short axes, respectively, in four different injections in bovine liver tissue not including intravasated material. The highest peak of optical imaging intensity was centered within the injection core.

In the analysis of ten regions within the sample from Fig. 12B, it was observed that optical imaging intensity for DOX was highest at the injection (Fig. 12C3, C4, and C6), gradually decreased with distance from the injection center (Fig. 12C3 to C12). Additionally, DOX distribution within vessels exhibited lower optical imaging intensities compared to the injection core (Fig. 12C3, C4, and C6).

## Discussion

The purpose of this study was to develop, optimize. and characterize gels that are imageable under US and x-ray/CT, utilizing POL as a potential drug carrier, such as for anti-cancer treatments. POL provided controlled drug release in vitro, even when mixed with the US, MBs, and iodixanol. The gel injectability was tested across various needle devices, showing that its distribution, both in 3D and 2D formats, were predictable and visible using US and CT in ex vivo and *postmortem* animal models.

Liao et al.^39^ conducted a similar study with varying POL concentrations from 2 to 17% and incorporated MBs in the gel. The authors reported a slight decrease of gelation temperature value compared to formulations without MBs. However, our gel and data showed the gelation temperature tended to increase with increased MB content. The stability of MBs was evidenced by optical micrographs of gels containing 1% and 10% of MBs at 37 °C. The addition of iodixanol, MBs, and DOX did not compromise the rheological properties of POL, which remained injectable due to its thixotropic nature (recovery of viscosity post stress), and quick transition from liquid to solid upon the cessation of shear stress. This property is crucial for ensuring that POL remains localized at the physiological temperature of 37°C^30^, maintaining its effectiveness for targeted therapy delivery across a variety of syringe sizes with associated differences in pressure and shear stress.

Prior work showed that POL resulted in 1.5-fold lower release rates of DOX over the initial 7 h under identical in vitro conditions compared to another formulation without POL^30^. This finding highlights the advantages of using a gel-based drug delivery system over a conventional percutaneous injection of a free drug.

TMP US studies showed that gels with 1 to 10% MB concentrations were visible, though visibility at 10% MB might be compromised due to higher concentration of MBs reflecting back the majority of the sound waves leading to acoustic shadowing in the far field. These studies helped assess the gel texture and acoustic properties. However, MBs concentrations over 1% led to acoustic artifacts like comet tails and gas shadowing, affecting visibility in the far field^46^. Therefore, to minimize such artifacts, MB concentration in gels was maintained below 1% for optimal imaging in ex vivo liver tissue. Although not designed for vessel embolization, the POL formulations might be used as an imageable embolic agent to occlude vessels. Full exploration of this potential application was beyond the scope of the work.

Adjusting POL's MB concentration from 0.001 to 0.1% resulted showing high localization and clarity in ex vivo liver without acoustic artifacts. The choice of % MBs was crucial to balance the acoustic signal for optimal contrast and minimal artifacts, while enhancing texture detail within the gel for better tissue structure and gel-tissue interface visualization. POL with 0.001% MBs showed greater detail and therefore higher entropy than with 0.01% MBs, suggesting a more detailed visualization of tissue structures within the gel. Entropy was used as a simplified method to characterize texture differences at the region of interest (POL deposited in tissue). However, clinical imaging of MBs employs advanced techniques such as power modulation and phase inversion to enhance the visualization of US contrast agents^47^. Contrast-Pulse-Sequence processes the reflections of US pulses to construct images of individual MBs, offering optimized contrast-to-tissue ratio^47^. These commercially available techniques for MBs-contrast-only provide enhanced details on MBs distribution in tissue, thereby improving imaging sensitivity and detection of tissue abnormalities, including those resulting tissue disruption after gel administration. Detailed Techniques for characterizing tissue disruption following gel administration were beyond the scope of this study and require further study.

The selection of needle delivery devices and techniques affected the visibility of POL during image-guided injections. POL injections, especially with MPIN needles, sometimes lost US visibility. This may be due to the high pressure and shear stresses exerted on the MBs during passage through the narrow gage needles, which leads their destruction, suggesting an increase in MBs concentration might be necessary for gel use with the MPIN. Another less critical factor might have been the multi-planar volumetric nature of MPIN injections, that may be more challenging to capture within one ultrasound volume. Nevertheless, in general, POL injections with 0.01% MBs showed consistent results across different needles, with gel dimensions and delivered volumes showing a linear relationship. This predictability, crucial in clinical settings, allows for precise definition of treatment areas under US, enhancing the translatability potential^48^.

For POL injections using MPIN, specific injection protocols of needle retractions and rotations may be needed to optimize reproducibility and desired therapeutic effect^49,50^. Despite reduced visibility under US, POL's deposition could still be monitored in real-time US without ionizing radiation. Generally, gel volumes under 1 mL are less visible than those above 1 mL.

As the gel is injected, lower volumes initially create a cavity within the liver parenchyma, via displacement of tissue. Subsequently, as the volume of the gel increases within the tissue, the gel exerts pressure outward from the cavity, filling the tissue pores. The gel remained localized during this injection process in normal liver. How this might extend to neoplastic tissue adjacent to normal liver remains undefined, but might be influenced by interstitial pressures, diffusion, interface of neoplasm with liver, and the underlying health of adjacent normal liver, e.g., cirrhosis or fatty infiltration.

Percutaneous gel injections can use radial or sequential intratumoral techniques^6,51,52^. For sequential injections, visualizing multiple injections simultaneously under US without interference is crucial. Five transducers studied showed that US visibility of adjacent POL depositions, ex vivo, varies with the beam array used. Curvilinear and microconvex arrays offer broader visualization but less superficial near field detail, whereas linear arrays provide detailed near-field views at the expense of a panoramic and far field penetration and perspective. Choosing the right transducer and optimizing imaging and acoustic parameters like frequency and gain are vital for high-quality images, with the choice also depending on the targets. Having an optimized setup of acoustic parameters and transducer may improve the visibility of any echo changes after gel injections. For this study, depending on the transducer, it was possible to distinguish changes in echotexture and tissue, potentially by formation of cavities filled with gel surrounded by tissue or pores or low-pressure channels filled with gel.

POL with DOX maintained US visibility, acoustic properties, and measured volume, showing stable ex vivo tissue visibility over 1.5 h. This stability may have value in applications requiring procedural monitoring of gel and drug dispersion, such as during multiple needle and catheter local procedures. However, actual visibility will likely vary under perfused in vivo conditions.

POL was successfully visualized using both US and x-ray/CT, showing consistent area, eccentricities, solidities, and dimensions in ex vivo and *postmortem* animal models for 1 mL and 3 mL injections. This demonstrated the potential for multimodal imaging in guiding intratumoral POL injections, akin to fusion guidance and combination multi-modality practices in biopsies and tumor ablations^40,41^. MBs and iodine as contrast agents were simultaneously incorporated into a hydrogel for potential use in multi-modality image-guided drug delivery.

There are some reports of contrast agents, such as for CT, US, magnetic resonance imaging, fluorescence, or single photon emission computed tomography, being incorporated in gels and tested in animal models for cancer therapy or tissue engineering^53,54^. In addition, MBs have been incorporated in POL for intratympanic applications^39^. Even with this plethora of formulations incorporating multimodal imaging gels, there is still a gap in gel formulation characterization using different needle devices, clinically relevant volumes, and commercial clinical instruments; as well as the incorporation of both MBs and iodine in the same gel.

In clinical practice, needle-based interventions may use US and CT alone or in combination for image guidance, depending on the needs for management of each patient as well as the preferences of the physician. The two modalities are based on distinctly different imaging physics and may provide different information, i.e., a lesion may be seen on one and not the other, as well as differential visualization of areas to be avoided during needle placement. The combination of the POL with MBs and iodixanol permits imaging with both modalities and facile switching between the two as needed for specific workflow needs at specific timepoints in patient care related to injectable therapeutics. Thus, the real time nature of US imaging can be used in conjunction with the intra-procedural CT scans as well with fusion imaging of both US and CT and may impact safe and accurate dosage and planned treatment volume target tissue localization, and perhaps clinical outcomes^55-60^.

The 3D morphology of US images for POL containing DOX was similar to that seen with CT imaging, suggesting US could be used to estimate and monitor drug delivery or treatment zones without ionizing radiation, offering a potentially cost-effective option in settings lacking CT or CBCT imaging. However, the volumes calculated from US 2D sweep were higher than that of CT, therefore, caution must be exercised given the slight disparity.

The investigation did not explore the potential for oscillation or cavitation of MBs to trigger drug release bursts, immunomodulation, inflammation or tissue destruction. The key factor for inducing cavitation with medical imaging probes is the Mechanical Index (MI) and its setting level. In this study, the MI was maintained low (1.2–1.4), and even at the highest setting, it would not induce inertial cavitation of the bubbles due to their presence in a viscous gel rather than in blood or aqueous solution^61,62^.

There is a potential for prolonged US imaging to induce minimal stable cavitation of the bubbles. However, distinguishing the effects of this in the release studies would be challenging compared to the natural release rate caused by micellar hydrolysis of the gel. In clinical US imaging scenarios involving this gel (with brief exposure), any effect from stable cavitation would likely be negligible compared to the natural release mechanisms of the gel itself.

The use of optical imaging to characterize DOX distribution in bovine liver tissue provided insights into the DOX distribution at various regions both at and distanced from the injection site. Additionally, optical imaging detected DOX intensity in intravasated material. Delgado et al. employed a similar method to characterize DOX in bovine liver tissue following injection of POL with DOX using SEHN, MSHN, and MPIN needle devices, yielding comparable profiles to those reported in this study^30^. In the present research, we expanded upon this information by examining DOX distribution within the injection site, including leaked material.

There were several limitations to this study. The study relied on in vitro, ex vivo, and post-mortem liver models that lack blood flow or tissue perfusion, which would influence the behavior of the POL delivery and subsequent drug release. However, the aim with several of these studies, i.e., effects of MB and iodixanol on drug release, were intended to establish the effects of the constituents on drug release rather than precise prediction of in vivo behavior. The differences in acoustic properties between liver and tumor tissues could not be addressed, nor were other factors such as blood flow, lymphatic flow, interstitial pressures, fascial planes, diffusion, convection, tissue heterogeneities or viscosity. As further considerations, the properties of the tumor and liver tissue may affect drug release and efflux, perhaps due to heterogeneous tissue properties, tumor fibrosis, liver cirrhosis, drug adsorption by tissue, or elevated interstitial pressure, either intrinsic to the tumor or induced by the gel injection. Thus, further testing in large animal models and appropriate tumor models is a requisite step in assessing appropriateness of this vector for clinical use such as for intratumoral hepatic injections. Such studies would assess POL delivery and drug elution with pharmacokinetic evaluations, providing better representation of clinical drug delivery performance.

## Conclusion

In this study, a gel based on POL visible under US and x-ray/CT was developed, optimized, and characterized in vitro,in ex vivo bovine liver, and in *postmortem* swine liver. The gel demonstrated sustained DOX release in vitro, unaffected by the presence of MBs, and was injectable through a variety of commercial needles due to its ability to recover viscosity after stress. Both US and x-ray/CT imageability were verified in ex vivo and *postmortem* analyses, and the gel was compatible with electromagnetic navigation for multimodal fusion imaging co-registration and co-display. The potentially predictable distribution of POL in *ex-vivo* and *postmortem* tissue enhances its translational potential for clinical use. Additionally, 3D US imaging allowed for monitoring and prediction of injected treatment volumes with fusion guidance without use of ionizing radiation. This research introduces a promising potential tool for image-guided intratumoral injections of anti-cancer agents.

## Experimental section

*The following materials were used in this study to develop and maintain the POL-based gel::* Poloxamer 407 (POL), purified, non-ionic (Sigma Aldrich, Inc; Saint Louis, MO, USA), Visipaque 320 (GE Healthcare, Waukesha, WI, USA), ultrapure agarose (ThermoFisher Scientific, Waltham, MA, USA), doxorubicin hydrochloride (DOX) (LC Laboratories, Woburn, MA, USA), 18:0 PC (DSPC) (Avanti lipids, Alabaster, Alabama, USA), propylene glycol, PEG 18 Stearate, chloroform, and glycerol (Sigma Aldrich, St. Louis, MO, USA), perfluorobutane gas (Matrix Scientific, Columbia, SC, USA). The needle devices were obtained as follows: Quadra-Fuse ST, 18G, 15 cm and Quadrafuse multi-pronged injection needles (Rex Medical, Conshohoken, PA, USA), Chiba biopsy needle, 18G, 10 cm, and Profusion needle, 19G, 15 cm (Cook Medical, Bloomington, IN, USA). Borosilicate vials, 8 mL, (Duran Wheaton Kimble, Millville, NJ, USA), silicon tubing 33 (Smiths Medical, Dublin OH, USA), and bovine livers (Balduccis Market, Bethesda, MD, USA).

*Microbubble preparation:* Microbubbles (MBs) were prepared as previously described by Owen et al.^63^ Briefly, 0.6 mL of DSPC (25 mg/mL) in chloroform and 0.4 mL of PEG-40-Stearate (10 mg/mL) were mixed in an 8 mL glass vial (Thomas Scientific, Swedesboro, NJ, USA). The organic solvent was evaporated under stirring overnight. The next day, the lipid film was dissolved in a solution consisting of 8:1:1 normal saline, glycerol, and polyethylene glycol, heated to 79 °C, and subsequently ultrasonicated (20 kHz for 2 min, 20% power) with a sonic probe (Q55 Qsonica Sonicators, Newtown, CT, USA) deep in the solution. After sonicating and collapsing the lipid structures, the probe tip was moved to the gas/liquid interface under perfluorobutane bubbling and, the solution was ultrasonicated (20 kHz for 20 s, 90% power). This produced a white solution of MBs, which was then rapidly cooled in ice water.

*Gel preparation:* As previously reported, POL formulation was prepared using the cold method.^28^ Briefly, POL powder was mixed with normal saline, and iodixanol (Visipaque 320) was added to achieve a concentration of 22% (w/v) POL and 40 mg/mL iodine in the formulation (POL). The mixture was then stirred at 4 °C for at least 12 h. To prepare POL formulation containing DOX, the above procedure was followed, and doxorubicin was subsequently added to achieve a final concentration of 10 mg/mL, and stirred for 5 days at 4 °C.

*Gel preparation with MBs:* MBs at a concentration of 1 × 10^9^ bubbles/mL^64^ were prepared in a saline:glycerol:polyethylene glycol mixture. The mixture was added via a pipette to the POL gel to produce concentrations ranging from 0.001 to 10% (v/v), where the volumetric ratio is the volume of the MBs mixture gel. After depositing the MBs, the solution was gently stirred to thoroughly mix the MBs with the POL gel. Due to the high viscosity and rapid gelation of POL gel, careful stirring was necessary to ensure even distribution of the MBs. For POL with DOX, the MBs were added to the gel product at 4 °C and the liquid material was shaken manually until the MBs were well dispersed. In most cases, MBs were used immediately after preparation, typically within one hour. Before each formulation was loaded into the syringe, the MBs were gently agitated manually and then mixed with POL.

*Optical microscopy:* MBs, POL, and POL with MBs were visualized using a fluorescence microscope (Imager M2, Zeiss, White Plains, NY, USA) with a 5X objective. MBs were examined using a hemocytometer and counted in MATLAB software (R2020a version, Mathworks, Natick, MA) using a method previously outlined.^64^ For the POL with MBs, 100 µl was added onto a microscope slide at room temperature. This was then observed under the optical microscope in one area. Subsequently, the slide with the bubble-gel was heated to 37 °C for 10 min, and the same area of the bubble-gel was observed again to determine if the MBs remained present after the temperature transition process from liquid to gel.

*Gelation time of POL with MBs:* Gelation times were estimated by the inverted tube method.^45^ Briefly, 400 µl of bubble-gel containing 0, 1, 3, 7, 9, and 10% of MBs (v/v) were added to borosilicate vials. Each vial was equilibrated at room temperature for 5 min and subsequently submerged in a 37 °C water bath. The vials were then inverted while still under water. The time at which the bubble-gel stopped flowing and remained at the bottom of the vial was recorded.

*Oscillatory rheology analysis:* The determination of the gelation temperatures for both imageable and non-imageable POL variants was conducted using a Discovery HR20 rheometer (TA Instruments, New Castle, DE) equipped with a 25.0 mm stainless steel parallel plate setup maintaining a 500 µm gap. Prior to conducting oscillatory rheology tests, both the parallel plate assembly and the stage holding the samples were coated with mineral oil (conforming to ASTM oil standards).

*Determination of gelation temperature:* The influence of increased MBs concentration (0, 0.01, 0.01, and 1%, v/v) on the gelation temperature of POL variants devoid of iodixanol, those incorporating iodixanol (40 mg/mL of iodine), and those containing DOX (10 mg/mL) was assessed following the methodology outlined by Baloglu, et al.^65^ This involved gradually heating the samples from 5 to 37 °C. During this process, the samples underwent a strain of 0.2% and an angular frequency of 6.0 rad/s. The gelation temperature was defined, in accordance with established literature convention^66,67^, as the midpoint of the storage modulus value (G’) from the temperature-dependent rheological data.

*Viscoelastic properties:* POL containing MBs concentration (0, 0.01, 0.01, and 1%, v/v), iodixanol (40 mg/mL of iodine), and DOX (10 mg/mL) was characterized for viscoelastic and thixotropic properties. A time sweep was performed for 1 h at 0.2% strain and 6 rad/s at 37 °C with a 1000% strain event applied for 1 min followed by a 60 min at 0.2% strain to evaluate G’ and G” values. The ƞ* was calculated as:

1 $$\eta * = \frac{{\sqrt {G^{\prime 2} + G^{\prime \prime 2} } }}{6rad/s}$$

ƞ* was noted right before, during, right after strain, 10 min after strain, and 56 min after strain. Percentage of ƞ* recovered was obtained from the G’ value during strain and G’ value right after strain. To determine the linear viscoelastic regions, the flow points of POL were determined with amplitude sweeps done from 0.1 to 1000% oscillation strain at 6 rad/s. Flow points were noted when G’ = G”, tan δ = 1. n = 3 for each measurement.

In vitro* drug release:* The in vitro drug release of POL with iodixanol (40 mg/mL), MBs concentration (0,0.01, 0.01, and 1%, v/v), and DOX (10 mg/mL) was evaluated with a dialysis cassette with a 3.5k molecular weight cut-off (Pur-A-Lyzer Midi 3500, Sigma Aldrich). Using infinite sink conditions, 800 µl of POL with DOX 10 mg/mL was added to the dialysis cassette and incubated at 37 °C for 10 min. The samples were then added to 50 mL conical tubes containing 30 mL of 37 °C normal saline and placed in a shaker (Roto-Therm Plus, Ward’s Science, Rochester, NY, USA) (rocking mode,10) maintained at 37 °C. 200 µl aliquots were taken at 0, 1, 2, 3, 4, 5, 6, 7, 21, 45, and 69 h with normal saline volume replacement. DOX concentration in each aliquot from POL was calculated from absorbance measurements with a microplate reader (Biotek Cytation 5, Agilent) at λ = 483 nm and by comparison to a calibration series of DOX solutions of known concentration.

*Tissue mimicking phantoms (TMP) US imaging:* A material reservoir measuring 4.5 × 11 × 4.5 cm was 3D printed (Ultimaker S3, New York, NY, USA) using tough black polylactic acid (PLA) (Ultimaker material, New York, NY, USA). The tissue mimicking phantoms were prepared with agarose 2%. Briefly, 0.5 g of agarose was added to 500 mL of deionized water and solubilized under microwave heating. The solubilized transparent liquid was transferred to the reservoir. Subsequently, 1 × 1 × 2 cm plastic cuvettes were inserted into the liquid agar to form cavities into which the POL could be placed. The solidified agarose was allowed to cool for 1 h at 4 °C. To assess the US B-mode imaging of the MBs and POL with MBs, the mean acoustic intensity was characterized by using a IU22 US system (Philips, Cambridge, MA, USA) with a C5-2 transducer for POL as a liquid (T = 21 °C) and POL as a gel (T = 37 °C), The transducer was positioned perpendicularly to the TMP and the imaging parameters were frequency = 77 Hz, 40%, and C = 54.

In addition to acoustic intensity, the acoustic heterogeneity of agarose-based TMP US images was obtained by determining the standard deviation of the gray-scale histogram pixel intensity of a rectangular region of interest (ROI) with 1 cm^2^ area. Any encountered artifacts were reported by manually delineating the area of the artifacts. These measurements were performed using Fiji Software^68^. The samples analyzed under these conditions were normal saline with 0, 1, 5, and 10% MBs and POL with the same concentrations of MBs. For imaging performed on solidified gels, the TMP containing the samples were incubated 1 h at 37 °C. Entropy, a statistical measure of randomness within the US signal that can be used to characterize the texture of the input image, was also determined utilizing 1 cm^2^ rectangular ROIs. Entropy of US images was acquired from MATLAB using e = entropy(I) function were I, is the US image imported into the MATLAB code. Finally, the area of US artifacts was manually measured using Fiji software.

*Vascular occlusion and dislodging of gel:* Rectangular prism-shaped agar phantoms (4.5 × 11 × 4.5 cm) with an empty cylindrical void (diameter = 0.3 cm; length = 12 cm) to model a vessel were fabricated using a black PVA 3D printed material (Herndon). POL containing 0, 0.1, 1, and 10% MBs was used to occlude the agar-vessel and equilibrated for 5 min at 37 °C. Subsequently, normal saline was passed using a 6 mL syringe with a rate of 1 mL/min while measuring pressure with a sensor (Omega Engineering, Inc, Norwalk, CT, USA). The pressure required to dislodge the gel was recorded.

Ex vivo* bovine modeling at physiological temperature:* The experiments were performed similarly as previously reported using an ex vivo bovine liver^69–72^. Briefly, 10 lb bovine livers (Balduccis Market, Bethesda, MD, USA) were submerged for two hours in 10 L of 1 × PBS at 37 C° (Gibco Thermo Fisher Scientific, Waltham, MA, USA). Once the liver reached equilibrium at 37 C°, formulations of POL containing MBs were injected.

Ex vivo* bovine liver injections of POL.* POL injections were performed using three needle devices: single end hole needle (SEHN, Cook), multiple side hole needle (MSHN, Cook) and multi-pronged injection needles (MPIN, deployed 1 cm, Rex Medical) (Table 3). Needles were guided using a 3D printed needle guide designed to be 45° (Herndon) or freehand, by an experienced radiologist, all with US imaging. The injections were performed with 12 mL syringes (Monoject, Dublin, OH, USA), or 6 mL high pressure syringes (Medallion, Merit Medical, South Jordan, UT, USA) for MPIN needles. Infusions of POL were done with an injection pump (Harvard Apparatus PhD Ultra Syringe Pump, Holliston, MA, USA).

**Table 3:** Summary of ex vivo bovine injections with POL exclusively monitored by B-mode US imaging.

Purpose	Formulations	Needle devices	Volume, Injection rate	US equipment	Imaging parameters	n
Assessment of imageability of gel upon MBs concentration	POL with 40 mg/mL of iodine and 0.001, 0.01, and 0.1% MBs	SEHN (Cook)	4 mL, 100 mL/h	Sonodynamics (Paris, France), curvilinear transducer	Gain = 51%, Frequency = 55 Hz	3
Assessment of imageability of gel upon needle devices	POL with 40 mg/mL of iodine and 0.01% MBs	SEHN (Cook), MSHN (Cook), and MPIN (Rex Medical)			Gain = 41%, Frequency = 40 Hz	SEHN (5), MSHN (4), MPIN (3)
Assessment of imageability of gel upon needle devices	POL with 40 mg/mL of iodine and 0.1% MBs	MPIN (Cook)			Gain = 50%, Frequency = 55 Hz	4

**Fig. S17**, shows the overall setup needed to perform ex vivo experiments.

From the obtained B-mode US images, measurements of major axis (equatorial distance), minor axis (meridian distance) and area of gels were determined with a customized MATLAB code. Acoustic intensities were acquired by using Fiji software by selecting ROIs manually. Textures such as acoustic heterogeneity and entropy were also obtained from MATLAB software with a customized code by selecting ROIs manually.

*Gel dynamics per mL of injected POL:* B-mode US images of POL injected, ex vivo, were analyzed using a curvilinear transducer and US equipment (Sonodynamics). POL, 4 mL, containing 0.01% MBs was injected with SEHN (Cook), MSHN (Cook), and MPIN (deployed 1 cm, Rex Medical) at 100 mL/h. The imaging parameters were: gain = 49%, and frequency = 56 Hz. Measurements of the major axis, minor axis, and area were obtained from MATLAB. In addition, eccentricity was calculated with the following formula:

2 $$Eccentricity=\frac{\sqrt{{Major axis}^{2}-{minor axis}^{2}}}{major axis}$$

Solidities were calculated with MATLAB, and the distance of the centroid of the injection to needle tip was determined manually using Fiji software.

*MPIN injection techniques:* POL was injected into ex vivo bovine liver with two injection techniques using MPIN (short-tip) (Rex Medical), and MPIN (regular tip) (Rex Medical). Technique one involved utilizing a short tip needle, where the prongs extended 2 cm. As the 4 mL volume was injected, the prongs were gradually pulled back in four steps of 0.5 cm each, retracting from 2 to 0.5 cm without rotating the needle device. Technique two employed a regular tip, advancing the prongs out to a length of 5 cm. Throughout the injection of 4 mL, the prongs were sequentially retracted in 1 cm increments, from 5 cm down to 1 cm, with approximately 0.8 mL administered per step. The device was not rotated during the process. During the injection techniques, the POL distribution was monitored with an EPIQ 7 US system (Philips) using a C5-1 curvilinear transducer and gain of 49%, and frequency of 62 Hz. Gel dynamics and analysis were performed as described above.

*Evaluation of US transducers in POL, *ex vivo*:* The US imageability of two 4 mL POL injections (10 mL/h) approximately 2 cm apart, as measured from the center of each injection, was evaluated with five transducers (Philips), C5-1 (curved array, aperture = 55.5mm, FOV = 111°, gain = 49%, frequency = 62 Hz), eL18-4 (linear array, gain = 62%, frequency = 67 Hz), X6-1 (volume sector, FOV = 100°, gain = 65%, frequency = 32 Hz), L12-3ERGO (Linear, aperture = 38mm, gain = 32%, frequency = 56 Hz), and L15-7io (Linear, aperture = 23mm, gain = 57%, frequency = 47 Hz). The texture of the obtained B-mode images was determined as described above using MATLAB.

*Gross pathology*: After injection, the ex vivo live was excised and the tissue was inspected to identify gel cavities formed. Tissue was also flash frozen in 2-mercaptoethanol (Aldrich) cooled in liquid nitrogen. A cryotome (Leica Biosystems, Deer Park, IL, USA) was used and set to obtain 100µm slices at -20 °C and subsequently observed in brightfield microscopy (Zeiss).

Ex vivo* bovine liver assessment of POL containing MBs and DOX*: 4 mL of POL injected at 10 mL/h with 0.01% MBs and 10 mg/mL of DOX was imaged in B-mode over 100 min. Images were taken every 10 min. The acoustic intensity and areas were determined over time using Fiji software.

*CBCT and fluoroscopy *ex vivo* imaging*: X-ray imaging was performed (Allura Xper FD20, Philips, Best, the Netherlands) with an imaging protocol of 120 kVp and 148 mA. Injections of 1 mL and 3 mL POL with 40 mg/mL of iodine from iodixanol were performed at 100 mL/h. X-ray imaging was compared with B-mode US imaging (Sonodynamics) using a curvilinear transducer (gain = 49%, frequency = 32 Hz). Lengths of the major and minor axes, area, eccentricity and solidity were determined with MATLAB as described previously in the ex vivo liver injections of POL section.

*Electromagnetic-based navigation system and US/CT image fusion*: A commercially available navigation system (EPIQ 7 PercuNav, Philips), was used for US image fusion with a CT scan (Brilliance MX8000, Philips, Cleveland, OH) acquired at 120 kVp, 300 mA and reconstructed as 2 mm slices at 1.5 mm intervals. The electromagnetic (EM) US tracking system consisted of an EM generator, EM tracked C5-1 probe, and patient reference. Multi-modality image fusion was performed for after injection of 4 mL of POL with 0.01% MBs and 40 mg/mL of iodine from iodixanol in an ex vivo bovine liver using a SEHN.

*3D US imaging*: Using the EM US tracking system described above, POL with 0.01% MBs, 40 mg/mL of iodine from iodixanol, and with and without 10 mg/mL of DOX was injected with MSHN (100 mL/h). While tracking the US transducer's C5-1 position, a sweep was performed across the region of interest. To build a 3D US reconstruction, a custom algorithm was used to associate the location of the transducer to each US image frame. The images were then stacked and oriented in 3D space and converted to DICOM format, in preparation for segmentation (3D Slicer, URL https://www.slicer.org/). A CT scan was acquired as above for comparison with 3D US imaging using MATLAB.

*Volumetric and morphometric analyses*: The solidity and sphericity of the 3D POL distributions from CT and US were determined using 3D Slicer for segmentation and Blender software (Blender Foundation, Amsterdam, Netherlands). The formulas used to calculate solidity and sphericity are as follows:

3 $$\text{Solidity}= \frac{V}{Convex hull volume}$$

4 $$Sphericity= \frac{{\pi }^\frac{1}{3}{\left(6V\right)}^\frac{2}{3}}{SA}$$

The 3D Slicer software was employed to measure the surface area and volume of the gel being injected. Blender was used to determine the convex hull volume, which refers to the smallest convex set that contains all the points of the gel injections' previously segmented volume via the thresholding of Hounsfield units. SA/V were also obtained after segmentation with both imaging modalities.

*CT and US postmortem swine liver imaging*: A female Yorkshire swine (52 kg) was euthanized following unrelated studies. After 30 min, the liver was manually injected with 4 mL of POL with 0.01% MBs, 40 mg/mL of iodine from iodixanol, and 10 mg/mL of DOX using a SEHN. The US (Epiq7) (gain = 60, frequency = 43 Hz) and CT images were obtained as described previously and were segmented using 3D Slicer to obtain 3D distribution.

2D imaging analysis for texture, area, major axis, minor axis, solidity and eccentricity were performed as previously noted and compared for both imaging modalities.

*DOX distribution in tissue with optical imaging:* to investigate the gel and the surrounding liver tissue following in situ gel deposition of DOX content, 4-mL with DOX (2mg/mL) were injected using a MSHN (n = 4) in ex vivo bovine liver at 10mL/h. Briefly, following in situ gel deposition the tissue was flash frozen and subsequently manually sliced to obtain 5mm tissue blocks. An In Vivo Imaging System (IVIS III, PerkinElmer, Waltham, MA) (λ_ex_ = 480 nm; λ_ex_ = 620 nm; Excitation time = 5s; Binning = small; F/stop = 1.2; FOV = E, and height = 1.5cm) was used to image tissue frozen blocks. DOX distribution profiles at the injection site were determined along a line throughout the center of the injection for equatorial and meridian axes using the Living Image 4.7.3 software (Perkin Elmer). The radiant efficiency values were normalized considering 1 as the maximum intensity and 0 as minimum intensity. In addition, to estimate DOX distribution surrounding tissue post-POL injection, seven tissue blocks of a sample (n = 1) with intravasated POLDOX were analyzed. Ten selected regions of this sample were characterized by drawing lines throughout the selected zones. The DOX intensity signals distributed in tissue were plotted using GraphPad Prism 9 software.

*Statistical analysis:* All experiments were done in triplicate unless otherwise noted. One-way ANOVA with post-Tukey test were done to obtain statistical differences between groups. T-test was performed for comparisons within two groups. Multivariable linear regression was used to calculate regression coefficients. Correlation matrices were obtained by computing Pearson correlation coefficients. GraphPad Prism 9 (GraphPad Software, Boston, MA, www.graphpad.com) was used for plots and statistical analyses.

## Supplementary Information

Supplementary Information.

## Data Availability

The datasets used and/or analysed during the current study available from the corresponding author on reasonable request.

## References

[1] International, W.C.R.F. Liver cancer statistics. Vol. 2022 (2020). <https://www.wcrf.org/cancer-trends/liver-cancer-statistics/#:~:text=liver%20cancer%20data-,Liver%20cancer%20is%20the%206th%20most%20common%20cancer%20worldwide.,of%20liver%20cancer%20in%202020

[2] Finn, R. S. *et al.* Atezolizumab plus bevacizumab in unresectable hepatocellular carcinoma. *N Engl. J. Med.* **382**, 1894–1905. 10.1056/NEJMoa1915745 (2020). 32402160 10.1056/NEJMoa1915745

[3] Cheng, A. L., Hsu, C., Chan, S. L., Choo, S. P. & Kudo, M. Challenges of combination therapy with immune checkpoint inhibitors for hepatocellular carcinoma. *J. Hepatol.* **72**, 307–319 (2020). 31954494 10.1016/j.jhep.2019.09.025

[4] Tapper, E. B. *et al.* Direct costs of care for hepatocellular carcinoma in patients with hepatitis C cirrhosis. *Cancer* **122**, 852–858. 10.1002/cncr.29855 (2016). 26716758 10.1002/cncr.29855

[5] Sheth, R. A. *et al.* Assessment of image-guided intratumoral delivery of immunotherapeutics in patients with cancer. *Jama Netw. Open* **3**(7), e207911 (2020). 32725245 10.1001/jamanetworkopen.2020.7911 PMC11984515

[6] Marabelle, A., Tselikas, L., De Baere, T. & Houot, R. Intratumoral immunotherapy: using the tumor as the remedy. *Annals of Oncology* **28**, xii33–xii43 (2017). 29253115 10.1093/annonc/mdx683

[7] Munoz, N. M. *et al.* Influence of injection technique, drug formulation and tumor microenvironment on intratumoral immunotherapy delivery and efficacy. *J Immunother Cancer* **9**, e001800 (2021). 33589526 10.1136/jitc-2020-001800 PMC7887346

[8] Cheong, L. W. S., Heng, P. W. S. & Wong, L. F. Relationship between polymer viscosity and drug release from a matrix system. *Pharm. Res.-Dordr.* **9**, 1510–1514. 10.1023/A:1015883501871 (1992). 10.1023/A:1015883501871 1475242

[9] Mikhail, A. S. *et al.* Hydrogel drug delivery systems for minimally invasive local immunotherapy of cancer. *Adv. Drug. Deliver. Rev.* **202**, 115083 (2023). 10.1016/j.addr.2023.115083 PMC11616795 37673217

[10] Wang, Q. Q., Tan, C., Qin, G. & Yao, S. K. Promising clinical applications of hydrogels associated with precise cancer treatment: A review. *Technol. Cancer Res. Treat.* **22**, 15330338221150322 (2023). 36604973 10.1177/15330338221150322 PMC9829993

[11] Chao, Y., Chen, Q. & Liu, Z. Smart injectable hydrogels for cancer immunotherapy. *Adv. Funct. Mater.*10.1002/adfm.201902785 (2020).33828443 10.1002/adfm.201902785

[12] Majumder, P. *et al.* Surface-fill hydrogel attenuates the oncogenic signature of complex anatomical surface cancer in a single application. *Nat. Nanotechnol.* **16**, 1251 (2021). 34556833 10.1038/s41565-021-00961-w PMC8595541

[13] Amiji, M. M., Lai, P. K., Shenoy, D. B. & Rao, M. Intratumoral administration of paclitaxel in an in situ gelling poloxamer 407 formulation. *Pharm. Dev. Technol.* **7**, 195–202 (2002). 12066574 10.1081/PDT-120003487

[14] Chung, C. K. *et al.* Doxorubicin loaded poloxamer thermosensitive hydrogels: Chemical, pharmacological and biological evaluation. *Molecules* **25**(9), 2219 (2020). 32397328 10.3390/molecules25092219 PMC7248767

[15] Fakhari, A. *et al.* Thermosensitive gel-based formulation for intratumoral delivery of toll-like receptor 7/8 dual agonist, MEDI9197. *J Pharm. Sci.* **106**, 2037–2045 (2017). 28456734 10.1016/j.xphs.2017.04.041

[16] Chung, C. K. *et al.* Thermosensitive hydrogels as sustained drug delivery system for CTLA-4 checkpoint blocking antibodies. *J. Control Release* **323**, 1–11 (2020). 32247805 10.1016/j.jconrel.2020.03.050

[17] Liu, Y. F. *et al.* pH-sensitive peptide hydrogels as a combination drug delivery system for cancer treatment. *Pharmaceutics* **14**(3), 652 (2022). 35336026 10.3390/pharmaceutics14030652 PMC8948763

[18] Anirudhan, T. S. & Mohan, A. M. Novel pH switchable gelatin based hydrogel for the controlled delivery of the anti cancer drug 5-fluorouracil. *Rsc Adv.* **4**, 12109–12118 (2014). 10.1039/c3ra47991a

[19] Jones, J. P., Sima, M., O’Hara, R. G. & Stewart, R. J. Water-borne endovascular embolics inspired by the undersea adhesive of marine sandcastle worms. *Adv. Healthc. Mater.* **5**, 795–801. 10.1002/adhm.201500825 (2016). 26806763 10.1002/adhm.201500825 PMC5703062

[20] Wang, D. S., Wagner, M., Butt, H. J. & Wu, S. Supramolecular hydrogels constructed by red-light-responsive host-guest interactions for photo-controlled protein release in deep tissue. *Soft Matter.* **11**, 7656–7662 (2015). 26292617 10.1039/C5SM01888A

[21] Jaiswal, M. K. *et al.* Thermoresponsive magnetic hydrogels as theranostic nanoconstructs. *Acs Appl. Mater. Interfaces* **6**, 6237–6247 (2014). 24716547 10.1021/am501067j PMC4025575

[22] Fakhari, A. & Anand Subramony, J. Engineered in-situ depot-forming hydrogels for intratumoral drug delivery. *J. Control Release* **220**, 465–475 (2015). 26585504 10.1016/j.jconrel.2015.11.014

[23] Brachi, G. *et al.* Intratumoral injection of hydrogel-embedded nanoparticles enhances retention in glioblastoma. *Nanoscale* **12**, 23838–23850 (2020). 33237080 10.1039/D0NR05053A PMC8062960

[24] Li, T. *et al.* Thermosensitive hydrogel co-loaded with gold nanoparticles and doxorubicin for effective chemoradiotherapy. *AAPS J.* **18**, 146–155. 10.1208/s12248-015-9828-3 (2016). 26381779 10.1208/s12248-015-9828-3 PMC4706291

[25] Ashrafi, K. *et al.* Characterization of a novel intrinsically radiopaque Drug-eluting Bead for image-guided therapy: DC Bead LUMI. *J. Control Release* **250**, 36–47 (2017). 28188808 10.1016/j.jconrel.2017.02.001 PMC5416940

[26] Negussie, A. H. *et al.* Synthesis and characterization of image-able polyvinyl alcohol microspheres for image-guided chemoembolization. *J. Mater. Sci. Mater. Med.* **26**, 1–10. 10.1007/s10856-015-5530-3 (2015). 10.1007/s10856-015-5530-3 PMC6663481 26105830

[27] Negussie, A. H. *et al.* Synthesis, characterization, and imaging of radiopaque bismuth beads for image-guided transarterial embolization. *Sci. Rep.* **11**, 533 (2021). 33436734 10.1038/s41598-020-79900-z PMC7804415

[28] Huang, L. *et al.* Thermo-sensitive composite hydrogels based on poloxamer 407 and alginate and their therapeutic effect in embolization in rabbit VX2 liver tumors. *Oncotarget* **7**, 73280–73291 (2016). 27602579 10.18632/oncotarget.11789 PMC5341979

[29] Dumortier, G., Grossiord, J. L., Agnely, F. & Chaumeil, J. C. A review of poloxamer 407 pharmaceutical and pharmacological characteristics. *Pharm. Res.-Dordr.* **23**, 2709–2728. 10.1007/s11095-006-9104-4 (2006). 10.1007/s11095-006-9104-4 17096184

[30] Delgado, J. F. *et al.* X-ray imageable, drug-loaded hydrogel that forms at body temperature for image-guided, needle-based locoregional drug delivery. *Sci. Rep.* **14**, 13352 (2024). 38858467 10.1038/s41598-024-64189-z PMC11164888

[31] Wells, P. N. T. & Liang, H. D. Medical ultrasound: imaging of soft tissue strain and elasticity. *J. R. Soc. Interface* **8**, 1521–1549 (2011). 21680780 10.1098/rsif.2011.0054 PMC3177611

[32] Chan, V. & Perlas, A. Basics of ultrasound imaging. *Atlas of ultrasound-guided procedures in interventional pain management*, 13–19 (2011).

[33] Nanda, N. C. History of echocardiographic contrast agents. *Clin. Cardiol.* **20**, I7–I11 (1997). 9383596 10.1002/clc.4960201304 PMC6655563

[34] Stride, E. Physical principles of microbubbles for ultrasound imaging and therapy. *Front. Neurol. Neurosc.* **36**, 11–22 (2015). 10.1159/000366223 25531659

[35] Leighton, T. *The Acoustic Bubble* (Academic Press Limited, 2012).

[36] Chai, S. C., Tan, P. J. & Tong, K. L. A review of the safety and clinical utility of contrast echocardiography. *Singap. Med. J.* **61**, 181–183 (2020). 10.11622/smedj.2019169 PMC7905138 31820006

[37] Leen, E. *et al.* Contrast-enhanced 3D ultrasound in the radiofrequency ablation of liver tumors. *World J. Gastroenterol.* **15**, 289–299 (2009). 19140228 10.3748/wjg.15.289 PMC2653325

[38] Lee, H. *et al.* Microbubbles used for contrast enhanced ultrasound and theragnosis: A review of principles to applications. *Biomed. Eng. Lett.* **7**, 59–69. 10.1007/s13534-017-0016-5 (2017). 30603152 10.1007/s13534-017-0016-5 PMC6208473

[39] Liao, A. H. *et al.* Development of thermosensitive poloxamer 407-based microbubble gel with ultrasound mediation for inner ear drug delivery. *Drug Deliv* **28**, 1256–1271. 10.1080/10717544.2021.1938758 (2021). 34142922 10.1080/10717544.2021.1938758 PMC8216251

[40] Abi-Jaoudeh, N. *et al.* Multimodality image fusion-guided procedures: technique, accuracy, and applications. *Cardiovasc. Inter. Rad.* **35**, 986–998. 10.1007/s00270-012-0446-5 (2012). 10.1007/s00270-012-0446-5 PMC3447988 22851166

[41] Wood, B. J. *et al.* Navigation systems for ablation. *J. Vasc. Intervent. Radiol.* **21**, S257–S263 (2010). 10.1016/j.jvir.2010.05.003 PMC2921632 20656236

[42] James, A. P. & Dasarathy, B. V. Medical image fusion: A survey of the state of the art. *Inform. Fusion* **19**, 4–19 (2014). 10.1016/j.inffus.2013.12.002

[43] Franz, A. M. *et al.* Electromagnetic tracking in medicine-a review of technology, validation, and applications. *IEEE T Med. Imag.* **33**, 1702–1725 (2014). 10.1109/TMI.2014.2321777 24816547

[44] Wood, B. J. *et al.* Navigation with electromagnetic tracking for interventional radiology procedures: A feasibility study. *J. Vasc. Intervent. Radiol.* **16**, 493–505 (2005). 10.1097/01.RVI.0000148827.62296.B4 PMC2375916 15802449

[45] Alonso, J. M., del Olmo, J. A., Gonzalez, R. P. & Saez-Martinez, V. Injectable hydrogels: From laboratory to industrialization. *Polymers* **13**, 650 (2021). 33671648 10.3390/polym13040650 PMC7926321

[46] Thickman, D. I., Ziskin, M. C., Goldenberg, N. J. & Linder, B. E. Clinical manifestations of the comet tail artifact. *J. Ultras. Med.* **2**, 225–230 (1983). 10.7863/jum.1983.2.5.225 6864869

[47] Pallwein, L. *et al.* Ultrasound of prostate cancer: Recent advances. *Eur Radiol* **18**, 707–715. 10.1007/s00330-007-0779-7 (2008). 17938936 10.1007/s00330-007-0779-7

[48] Austin, C. P. Opportunities and challenges in translational science. *Cts-Clin. Transl. Sci.* **14**, 1629–1647. 10.1111/cts.13055 (2021). 10.1111/cts.13055 PMC8504824 33982407

[49] Sudheendra, D. *et al.* Comparison of three different needles for percutaneous injections. *Cardiovasc. Inter. Rad.* **30**, 151–152. 10.1007/s00270-005-0387-3 (2007). 10.1007/s00270-005-0387-3 PMC2555975 16988874

[50] Amalou, H. & Wood, B. J. Intratumoral Gene Therapy Injections with a Multipronged, Multi-Side Hole Needle for Rectal Carcinoma. *Cardiovasc. Inter. Rad.* **36**, 561–562. 10.1007/s00270-012-0396-y (2013). 10.1007/s00270-012-0396-y PMC3532565 22535244

[51] Champiat, S. *et al.* Intratumoral Immunotherapy: From Trial Design to Clinical Practice. *Clin. Cancer Res.* **27**, 665–679 (2021). 32943460 10.1158/1078-0432.CCR-20-0473

[52] Tselikas, L. *et al.* Interventional radiology for local immunotherapy in oncology. *Clin. Cancer Res.* **27**, 2698–2705 (2021). 33419781 10.1158/1078-0432.CCR-19-4073

[53] Dong, Y. C., Bouche, M., Uman, S., Burdick, J. A. & Cormode, D. P. Detecting and monitoring hydrogels with medical imaging. *Acs Biomater. Sci. Eng.* **7**, 4027–4047 (2021). 33979137 10.1021/acsbiomaterials.0c01547 PMC8440385

[54] Lemdani, K. *et al.* Mucoadhesive thermosensitive hydrogel for the intra-tumoral delivery of immunomodulatory agents, evidence of adhesion by means of non-invasive imaging techniques. *Int. J. Pharm.* **567**, 11841 (2019). 10.1016/j.ijpharm.2019.06.012 31176849

[55] Charboneau, J. W., Reading, C. C. & Welch, T. J. CT and sonographically guided needle biopsy: current techniques and new innovations. *AJR Am. J. Roentgenol.* **154**, 1–10. 10.2214/ajr.154.1.2104689 (1990). 2104689 10.2214/ajr.154.1.2104689

[56] Mauri, G. *et al.* Real-time US-CT/MRI image fusion for guidance of thermal ablation of liver tumors undetectable with US: Results in 295 cases. *Cardiovasc. Inter. Rad.* **38**, 143–151. 10.1007/s00270-014-0897-y (2015). 10.1007/s00270-014-0897-y 24806953

[57] Lee, M. W. *et al.* Targeted sonography for small hepatocellular carcinoma discovered by CT or MRI: Factors affecting sonographic detection. *Am. J. Roentgenol.* **194**, W396–W406 (2010). 20410384 10.2214/AJR.09.3171

[58] Kim, P. N. *et al.* Planning ultrasound for percutaneous radiofrequency ablation to treat small (<= 3 cm) hepatocellular carcinomas detected on computed tomography or magnetic resonance imaging: a multicenter prospective study to assess factors affecting ultrasound visibility. *J. Vasc. Intervent. Radiol.* **23**, 627–634 (2012). 10.1016/j.jvir.2011.12.026 22387030

[59] Boone, J. M., Hendee, W. R., McNitt-Gray, M. F. & Seltzer, S. E. Radiation exposure from CT scans: how to close our knowledge gaps, monitor and safeguard exposure-proceedings and recommendations of the radiation dose summit, sponsored by NIBIB, February 24–25, 2011. *Radiology* **265**, 544–554 (2012). 22966066 10.1148/radiol.12112201 PMC3480815

[60] Davenport, M. S. *et al.* Use of intravenous iodinated contrast media in patients with kidney disease: Consensus statements from the american college of radiology and the national kidney foundation. *Radiology* **294**, 660–668. 10.1148/radiol.2019192094 (2020). 31961246 10.1148/radiol.2019192094

[61] Wei, H. Q., Liu, J. H., Wang, W. N., Qiu, C. H. & Hu, Z. W. Effect of diagnostic ultrasound at different mechanical indexes on microbubbles. *Int. J. Clin. Exp. Med.* **12**, 12591–12597 (2019).

[62] Luo, J., Xu, W. L., Zhai, Y. W. & Zhang, Q. Experimental study on the mesoscale causes of the influence of viscosity on material erosion in a cavitation field. *Ultrason. Sonochem.* **59**, 104699 (2019). 31476699 10.1016/j.ultsonch.2019.104699

[63] Owen, J. *et al.* A versatile method for the preparation of particle-loaded microbubbles for multimodality imaging and targeted drug delivery. *Drug Deliv. Transl. Res.* **8**, 342–356. 10.1007/s13346-017-0366-7 (2018). 28299722 10.1007/s13346-017-0366-7 PMC5830459

[64] Sennoga, C. A. *et al.* On sizing and counting of microbubbles using optical microscopy. *Ultrasound Med. Biol.* **36**, 2093–2096 (2010). 21030137 10.1016/j.ultrasmedbio.2010.09.004

[65] Baloglu, E., Karavana, S. Y., Senyigit, Z. A. & Guneri, T. Rheological and mechanical properties of poloxamer mixtures as a mucoadhesive gel base. *Pharm. Dev. Technol.* **16**, 627–636. 10.3109/10837450.2010.508074 (2011). 20715905 10.3109/10837450.2010.508074

[66] Edsman, K., Carlfors, J. & Petersson, R. Rheological evaluation of poloxamer as an in situ gel for ophthalmic use. *Eur. J. Pharm. Sci.* **6**, 105–112 (1998). 9795025 10.1016/S0928-0987(97)00075-4

[67] Russo, J., Fiegel, J. & Brogden, N. K. Rheological and drug delivery characteristics of poloxamer-based diclofenac sodium formulations for chronic wound site analgesia. *Pharmaceutics* **12**, 1214 (2020). 33333773 10.3390/pharmaceutics12121214 PMC7765230

[68] Schindelin, J. *et al.* Fiji: an open-source platform for biological-image analysis. *Nat. Methods* **9**, 676–682 (2012). 22743772 10.1038/nmeth.2019 PMC3855844

[69] Delgado, J. *et al.* Abstract No. 552 Dual ultrasound/x-ray imageable thermosensitive gel for intratumoral drug delivery and vessel embolization. *J. Vasc. Intervent. Radiol.* **33**, S205 (2022).

[70] Delgado, J. *et al.* Abstract No. 551 Characterization of an x-ray-imageable gel for image-guided intra-tumoral drug injections. *J. Vasc. Intervent. Radiol.* **33**, S205 (2022).

[71] Delgado, J. *et al.* Abstract No. 242 distribution of imageable thermosensitive drug-loaded gel in ex vivo bovine liver depends on needle type and injection technique. *J. Vasc. Intervent. Radiol.* **34**, S110 (2023). 10.1016/j.jvir.2022.12.305

[72] Varble, N. A. *et al.* Morphometric characterization and temporal temperature measurements during hepatic microwave ablation in swine. *PLoS One* **18**, e0289674 (2023). 37540658 10.1371/journal.pone.0289674 PMC10403086

