# Supplementary material for: Ultrasound and x-ray imageable poloxamer-based hydrogel for loco-regional therapy delivery in the liver
Source: Sci Rep. 2024 Sep 3;14:20455. doi: 10.1038/s41598-024-70992-5 (PMC11372101; doi:10.1038/s41598-024-70992-5)
Supplement: Supplementary file 1 — Supplementary Information. [file 41598_2024_70992_MOESM1_ESM.docx]

**Results**

**Development and Characterization of POL containing Microbubbles**

|  |
| --- |
| **Fig.S1.** Characterization of microbubble-gels with gelation time and optical microscopy assessment. (A) Size distribution of MBs dispersed in normal saline with optical microscopy (inset). (B) Gelation times of microbubble-gels containing MBs ranging from 0% to 10% (v/v). (C), (E), and G) depict bubble-gels with 0%, 1%, and 10% MBs (v/v), respectively, at 21 °C (room temperature). (D), (F), and (H) depict bubble-gels with 0%, 1%, and 10% MBs (v/v), respectively, at 37 °C (body temperature). *, p=0.0185; **, p=0.004; ns, p=0.999). |

**Gelation temperatures.**

The gelation temperatures of POL changed after adding MBs (**Fig. S2**). The gelation temperatures were: 22.2 +0.3°C for 0% MBs, 22.2±0.1°C for 0.01% MBs, 22.5±0.0°C for 0.1% MBs, and 23.8±0.5°C for 1% MBs. Statistically significant differences were observed between the formulations with 0% MBs and 1% MBs (p=0.0008), 0.01% MBs and 1% MBs (p=0.0009), and 0.1% MBs and 1% MBs (p=0.0031). For formulations of POL containing MBs with iodixanol, the gelation temperatures were 31.6 +0.5°C, 32.6±0.2°C, 22.2±0.2°C, and 24.4±0.3°C for 0%, 0.01%, 0.1%, and 1% MBs, respectively. Comparisons between formulations showed statistical differences: 0 % and 0.01%MBs (p=0.0378), 0 % and 0.1% MBs; 0 % and 1% MBs; 0.01% and 0.1%MBs; 0.01%, and 1% MBs (p<0.0001), 0.1% and 1% MBs (p=0.0002).

|  |
| --- |
| **Fig. S2.** (A)G’ and G” temperature ramp from 5°C to 37 °C of POL formulations containing MBs, and (B). MBs with iodixanol. |

|  |
| --- |
| **Fig. S3.** G’, and G” values of POL containing MBs, iodixanol, and DOX. |

**Viscoelastic characteristics.**

At a temperature of 37°C, the POL formulation with 0.01% MBs demonstrated a G’ that was 61.3+3 times greater than its loss modulus (G”). For the POL containing 0.1% MBs, the storage modulus was 50+4.2 times higher than the loss modulus. In the case of the 1% MBs POL, the storage modulus was 29.2+4.8 times greater than the loss modulus. The ƞ* for each POL were 6174.1+88.3 Pas for 0.01% MBs, 6044.9+138.4 Pas for 0.1% MBs, and 6262.2 +586.6 Pas for 1% MBs. The addition of MBs to the polymeric system decreased G’. The G’ of 0% MBs POL formulation was 1.1+0.0 (p=0.0026), 1.2+0.0 (p=0.0004), and 1.1+0.1 (p=0.0088) times higher than 0.01, 0.1, and 1% MBs respectively.

Upon the addition of iodixanol into the POL formulations containing MBs, the viscoelastic properties tended to increase G’ as %MBs also increased. The G’ values for these formulations were: for the 0% MBs, the G’ was 5517.6 +209.17 Pa; for the 0.01% MBs concentration, G' was 6443.1±50.6 Pa; for the 0.1% concentration, G' significantly increased to 16215.2 Pa; and for the 1% concentration, G' was 14260.1±1645.5 Pa. After comparing the G’ of 0% MBs, the values were significantly lower than 0.1%, and 1% MBs (p<0.0001). Statistically, the G' of the 0.01% MBs formulation was significantly lower compared to the 0.1% and 1% MBs formulations (p<0.0001). However, the difference in G' between the 0.1% and 1% MBs formulations was less pronounced (p=0.0060). The ƞ* of 0.01%, 0.1% and 1%MBs were 1.1+0.0,2.9+0.1, and 2.5+0.3 times higher than 0% MBs. Additionally, the ƞ* 1% MBs was 2.2+0.3 times higher than 0.01%.

|  |
| --- |
| **Fig. S4.** A. Viscoelastic properties of POL containing MBs and B. MBs with iodixanol. **p<0.01, ***p<0.001, ****p<0.0001. |

**Recovery of G’ after a high strain event.**

**Table S1** summarized the ƞ* of POL formulations containing MBs without iodixanol, with iodixanol and with iodixanol and DOX (10mg/mL), as well as their % of ƞ* recoved after strain. Interestingly, addition of DOX to the formulations increased its % of ƞ* recovered after strain compared to POL with and without iodixanol and then compared p values are included in **Table S2**.

|  |
| --- |
| **Fig. S5.** Thixotropic properties of POL without iodixanol, containing 0.01% (A), 0.1% (B), and 1% (C) MBs. |

|  |
| --- |
| **Fig. S6.** Thixotropic properties of POL with iodixanol (40mg/mL of iodine), containing 0.01% (A), 0.1% (B), and 1% (C) MBs. |

|  |
| --- |
| **Fig. S7.** Thixotropic properties of POL with iodixanol (40mg/mL of iodine) and DOX (10mg/mL), containing 0.1% (A), 1% (B) MBs. |

| **Table S1.** Complex viscosities η* of POL formulations before, during and after a high strain event with its % of η* recovered right after high strain. | | | | | | |
| --- | --- | --- | --- | --- | --- | --- |
|  | η*, Before strain | η*, During strain | η*, Right after strain | η*,10min after strain | η*,56min after strain | % of η* recovered |
| Sample |  |  |  |  |  |  |
| POL + 0.01% MBs | 6174.1 +88.3 | 16.8 +0.1 | 4277.6 +74.9 | 4725.0 +85.4 | 4947.0 +49.5 | 69.3 +0.7 |
| POL + 0.1% MBs | 6044.9 +138.1 | 16.9 +0.3 | 3837.3 +109.2 | 4318.3 +146.6 | 4602.3 +200.5 | 63.5 +1.5 |
| POL + 1% MBs | 6262.2 +586.6 | 17.0 +1.6 | 4312.3 +373.9 | 4790.5 +419.6 | 5039.6 +491.2 | 68.9 +0.5 |
| POL + Iodixanol + 0.01% MBs | 2656.9 +26.0 | 5.8 +0.5 | 1161.8 +37.8 | 2301.2 +262.9 | 2557.2 +98.6 | 43.7 +1.0 |
| POL + Iodixanol + 0.1% MBs | 6621.2 +121.2 | 18.7 +0.3 | 4154.5 +139.1 | 4618.7 +145.8 | 4871.5 +124.1 | 62.7 +1.0 |
| POL + Iodixanol + 1% MBs | 5824.0 +672.2 | 15.6 +1.5 | 3726.7 +511.0 | 4184.6 +577.6 | 4420.2 +573.9 | 63.9 +2.0 |
| POL + Iodixanol + DOX + 0.01% MBs | 4759.5 +146.3 | 14.8 +0.2 | 3652.7 +54.3 | 4087.7 +67.2 | 4245.4 +80.2 | 76.8 +1.8 |
| POL + Iodixanol + DOX + 0.1% MBs | 4854.4 +371.8 | 15.2 +1.1 | 3830.5 +231.5 | 4276.1 +242.0 | 4456.4 +256.2 | 79.0 +1.4 |
| POL + Iodixanol + DOX + 1% MBs | 7691.3 +713.7 | 23.4 +1.8 | 5603.1 +281.0 | 6205.6 +353.1 | 6639.6 +606.4 | 73.1 +3.7 |

| **Table S2.** P values of the comparison of several % of η* recovered POL formulations right after high strain. |  | |
| --- | --- | --- |
| Sample | p values | |
| POL + 0.01% MBs vs. POL + Iodixanol + 1% MBs | * | 0.0307 |
| POL + 0.01% MBs vs. POL + Iodixanol + DOX + 0.01%MBs | ** | 0.0015 |
| POL + 0.01% MBs vs. POL + Iodixanol + DOX + 0.1% MBs | **** | <0.0001 |
| POL + 0.01% MBs vs. POL + Iodixanol + DOX + 1% MBs | ns | 0.2430 |
| POL + 0.1% MBs vs. POL + 1% MBs | * | 0.0300 |
| POL + 0.1% MBs vs. POL + Iodixanol + 0.01% MBs | **** | <0.0001 |
| POL + 0.1% MBs vs. POL + Iodixanol + 0.1% MBs | ns | 0.9998 |
| POL + 0.1% MBs vs. POL + Iodixanol + 1% MBs | ns | >0.9999 |
| POL + 0.1% MBs vs. POL + Iodixanol + DOX + 0.01% MBs | **** | <0.0001 |
| POL + 0.1% MBs vs. POL + Iodixanol + DOX + 0.1% MBs | **** | <0.0001 |
| POL + 0.1% MBs vs. POL + Iodixanol + DOX + 1% MBs | **** | <0.0001 |
| POL + 1% MBs vs. POL + Iodixanol + 0.01% MBs | **** | <0.0001 |
| POL + 1% MBs vs. POL + Iodixanol + 0.1% MBs | * | 0.0103 |
| POL + 1% MBs vs. POL + Iodixanol + 1% MBs | ns | 0.0523 |
| POL + 1% MBs vs. POL + Iodixanol + DOX + 0.01% MBs | *** | 0.0009 |
| POL + 1% MBs vs. POL + Iodixanol + DOX + 0.1% MBs | **** | <0.0001 |
| POL + 1% MBs vs. POL + Iodixanol + DOX + 1% MBs | ns | 0.1553 |
| POL + Iodixanol + 0.01% MBs vs. POL + Iodixanol + 0.1% MBs | **** | <0.0001 |
| POL + Iodixanol + 0.01% MBs vs. POL + Iodixanol + 1% MBs | **** | <0.0001 |
| POL + Iodixanol + 0.01% MBs vs. POL + Iodixanol + DOX + 0.01% MBs | **** | <0.0001 |
| POL + Iodixanol + 0.01% MBs vs. POL + Iodixanol + DOX + 0.1% MBs | **** | <0.0001 |
| POL + Iodixanol + 0.01% MBs vs. POL + Iodixanol + DOX + 1% MBs | **** | <0.0001 |
| POL + Iodixanol + 0.1% MBs vs. POL + Iodixanol + 1% MBs | ns | 0.9953 |
| POL + Iodixanol + 0.1% MBs vs. POL + Iodixanol + DOX + 0.01% MBs | **** | <0.0001 |
| POL + Iodixanol + 0.1% MBs vs. POL + Iodixanol + DOX + 0.1% MBs | **** | <0.0001 |
| POL + Iodixanol + 0.1% MBs vs. POL + Iodixanol + DOX + 1% MBs | **** | <0.0001 |
| POL + Iodixanol + 1% MBs vs. POL + Iodixanol + DOX + 0.01% MBs | **** | <0.0001 |
| POL + Iodixanol + 1% MBs vs. POL + Iodixanol + DOX + 0.1% MBs | **** | <0.0001 |
| POL + Iodixanol + 1% MBs vs. POL + Iodixanol + DOX + 1% MBs | *** | 0.0001 |
| POL + Iodixanol + DOX + 0.01% MBs vs. POL + Iodixanol + DOX + 0.1% MBs | ns | 0.8300 |
| POL + Iodixanol + DOX + 0.01% MBs vs. POL + Iodixanol + DOX + 1% MBs | ns | 0.2564 |
| POL + Iodixanol + DOX + 0.1% MBs vs. POL + Iodixanol + DOX + 1% MBs | * | 0.0146 |

**Flow point**

The flow points of POL without iodixanol and DOX were: 4.8 +0.7,5.6+0, 5.6+0, and 6.0+0.8 for 0,0.01,0.1, and 1% MBs. There was not statistical significance encountered when compared all formulations. When POL containing iodixanol were compared, the flow point of POL with 0.1% MBs was the highest compared to 0%, 0.01% and 1% MBs (p<0.0001; p<0.0001; p=0.0004, respectively). The flow points of POL containing iodixanol were: 3.6+1.2, 3.2+0.4, 8.7+0, 5.5+0 for 0,0.01,0.1, and 1% MBs, respectively. Adding DOX to POL containing iodixanol resulted in the following flow points 6.0+0.8, 4.4+0, 4.4+0, 6.1+0.8 for 0,0.01,0.1, and 1% MBs, respectively, making 1% MBs the highest compared to 0.01 and 0.1% MBs (p=0.0382 for both).

|  |
| --- |
| **Fig.S8.** G’ and G” as a function of oscillation strain at 37 °C for POL (A) not containing iodixanol, (B) containing iodixanol, and (C) DOX for 0%, 0.01%, 0.1%, and 1% MBs. |

**G’ and G” as a function of frequency sweep**

|  |
| --- |
| **Fig.S9.** G’ and G” as a function of frequency sweep at 37 °C for POL (A) not containing iodixanol, (B) containing iodixanol, and (C) DOX for 0%, 0.01%, 0.1%, and 1% MBs. |

**Ultrasound imaging assessment with Tissue Mimicking Phantoms (TMP).**

Acoustic intensity in TMP will change depending on the presence or absence of POL and depending on the liquid or gel state of POL (**Fig.S10**). The acoustic intensity of POL with iodixanol alone compared with normal saline had low average pixel intensity and detectability via B-mode (p>0.9999) (**Fig.S10A and S10B**). With MBs added to POL at 1% (v/v), the whole of POL was detectable via ultrasound imaging with a high average pixel intensity relative to the background level (**Fig. S10E-2H and Fig.S10 I toS10L**). The acoustic intensity will depend on the %MBs and the liquid or solid state of POL (**Fig. S10M**). For the case of 1%MBs formulations, the acoustic intensity increased with the addition of POL compared to normal saline without POL (1%MBs (without POL) vs POL 1% MBs (liquid), p<0.0001; and (1%MBs (without POL) vs POL 1% MBs (gel) p<0.0001). The acoustic intensity of formulations with 5% MBs in normal saline were not statistically significant when compared with POL (liquid) and POL (gel) (p>0.9999). Adding 10%MBs to the formulations enhanced the acoustic intensity and was similar as 1%MBs formulations since POL (gel) enhanced the acoustic intensity compared to POL (liquid) (p<0.0001) and normal saline with 10% MBs (p<0.0001).

The acoustic heterogeneity increased by augmenting %MBs in liquid formulations and decreased in gel form formulations (**Fig. S10N**). The acoustic heterogeneity of 0,1,5, and 10%MBs without POL were 0.6+0.3, 79.3+16.1, 122.1+10.8, and 125.0+6.1 a.u respectively with a linearly increase behavior (y=3.2x + 20.9, R^2^=0.9). POL (liquid) containing 0,1,5, and 10%MBs were 3.1+1.9, 151.7+4.4, 123.4+5.7, and 133.2+7.8 a.u respectively with a linearly increase behavior (y=2.4x + 19.3, R^2^=1.0). Finally, the acoustic heterogeneity for POL (gel) was 0.7+0.3, 143.5+9.1, 119.5+6.7, and 154.0+7.0 a.u for 0,1,5, and 10%MBs with a linearly decreasing behavior (y=-4.4x + 33.3, R^2^=0.9). Formulation with 1%MBs of gel POL were higher than 10%MBs of gel POL (p<0.0001), in addition, formulation with 5%MBs of gel POL were higher than 10%MBs (p<0.0055).

The entropy of ultrasound images calculated from the agar phantoms changed depending on the %MBs, the presence or absence of POL and liquid or gel state of POL (**Fig. S10O**). Formulations with 1%MBs had entropies of 6.9+0.2, 6.9+0.2, and 7.0+0.1 a.u for no gel, liquid POL and gel POL respectively. Formulations with 5%MBs had entropies of 7.0+0.2, 6.6+0.1, and 6.9+0.1 a.u for no gel, liquid POL and gel POL respectively. Finally, formulations with 10% MBs had entropies of 7.1+0.2, 7.5+0.3, and 6.7+0.1 a.u for no gel, liquid POL and gel POL respectively. When comparing formulations with 1,5, and 10% MBs of gel POL, there were no statistical significance (p>0.1).

The acoustic artifacts such as comet tails were measured for TMP for samples with different %MBs in terms of area (**Fig. S10P**). Comet tails were defined as the acoustic intensity present below the rectangular wells where the sample is located in the ultrasound image, which resembles a blurred bright tail. In general, the sample with higher area of acoustic artifacts are samples beyond 1% MBs (5%, and 10% MBs). For example, the area of the comet tail of 10%MBs in liquid form was 1.96 cm^2^+0.1 and was higher than 10%MBs of gel POL (p<0.0001). Interestingly, the sample without acoustic artifacts were POL in gel form with 1% MBs.

Because POL with 1%MBs provided high acoustic intensity compared to the background and there were no detectable acoustic artifacts in TMP, formulations of POL below 1%MBs were selected for *ex vivo* experiments.

|  | **0%MBs** | **1%MBs** | **5%MBs** | **10%MBs** |
| --- | --- | --- | --- | --- |
|  | **A** | **B** | **C** | **D** |
| **Control**  **MBs (no gel)** | **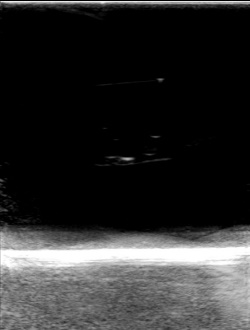** | **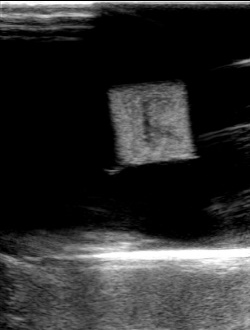** | **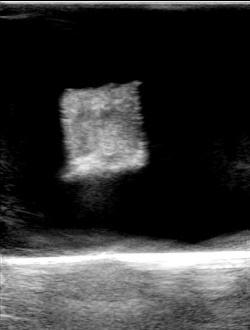** | **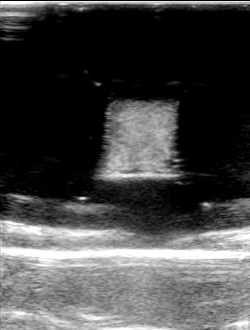** |
|  | **E** | **F** | **G** | **H** |
| **POL (Liquid)** | **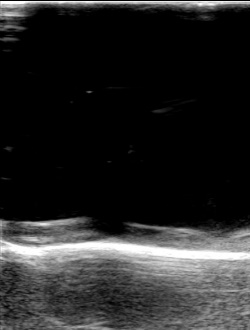** | **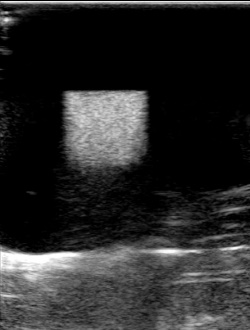** | **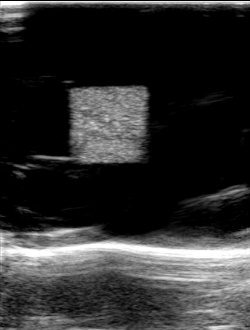** | **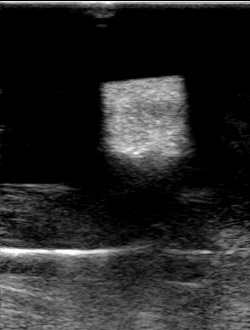** |
|  | **I** | **J** | **K** | **L** |
| **POL (gel)** | **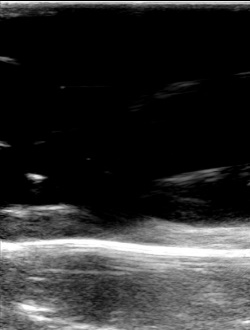** | **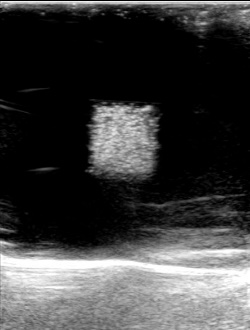** | **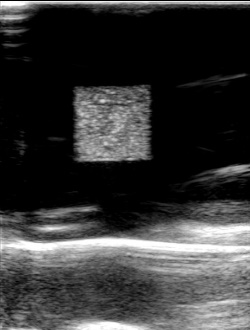** | **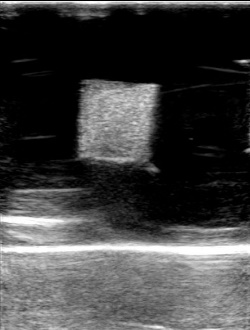** |
|  |  |  |  |  |
|  | | | | |
| **Fig. S10.** Acoustic assessment of POL with MBs and controls (No POL) in TMP. B-mode images of controls: normal saline (0%MBs) (A), 1%MBs(B), 5%MBs(C), and 10%MBs(D). B-mode images of POL in liquid form containing MBs: 0%(E), 1%(F), 5%(G), and 10%MBs(H). B-mode images of POL in gel form containing MBs: 0%(I), 1%(J), 5%(K), and 10%MBs(L). Acoustic intensity (M), acoustic heterogeneity (N), and area of acoustic artifacts (O) of POL with MBs and controls. *p<0.05, **p<0.01, ***p<0.001, ****p<0.0001. | | | | |

**Assessment of POL extravasation with agarose-based vessel phantom.**

In an event of vessel extravasation post-injection, the ability of POL to remain localized was assessed by measuring the pressure to de-occlude an agar-based vessel filled with POL with different %MBs (**Fig. S11**). The measured pressures to de-occlude the agar-based vessel were 2.4+0.6, 2.5+0.3, 2.1+0.5, and 1.6+0.3 bars for 0, 0.1, 1, and 10% MBs, although not statistically significant (p>0.09).

|  |
| --- |
| **Fig. S11.** Pressure needed to de-occlude an agar-based mimicking vessel filled with POL with different %MBs. |

**Assessment of ultrasound imageability of POL in *ex vivo* tissue.**

POL containing from 0.001%MBs up to 0.1% MBs was imageable under ultrasound without acoustic artifacts after injection (4mL, 100mL/h) in *ex vivo* bovine tissue (**Fig. S12A to S12C**). Clear and bright depositions of imageable POL were obtained in *ex vivo* bovine livers with a high degree of localization (**Fig. S12A to S12C**). The acoustic intensity of the injected POL with single end-hole needle (SEHN) was 130.5+2.6, 143.8+24.3, and 156.5+20.0 a.u for 0.001%, 0.01%, and 0.1% MBs with no difference among them (p>0.26) (**Fig. S12D**). The acoustic heterogeneity was 24.5+2.7 a.u., 19.5+2.7 a.u., and 21.5+5.3 a.u for 0.001%, 0.01%, and 0.1% MBs respectively. There were no differences in acoustic heterogeneity for each tested formulation (p>0.3) (**Fig. S12E**). The entropy of the formulations was 6.9+0.3, 6.3+0.0, and 6.5+0.2 a.u for 0.001%, 0.01%, and 0.1% MBs respectively (**Fig. S12F**). The area of the injected material was similar across the tested formulations with 1.6+0.5, 2.4+0, and 1.6+0.2 cm^2^ for 0.001%, 0.01%, and 0.1% MBs (**Fig. S12G**). These results demonstrate the reproducibility of the imageability and the area of treatment after injection of POL under ultrasound with the tested % MBs.

| **A**  **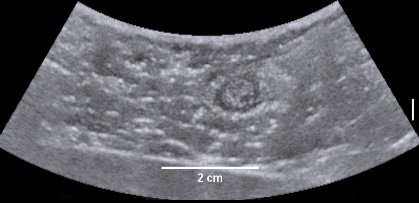** | **B**  **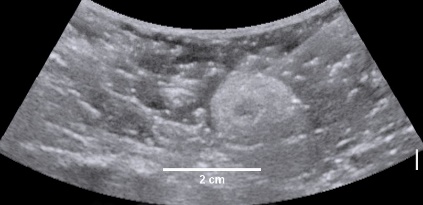** | **C**  **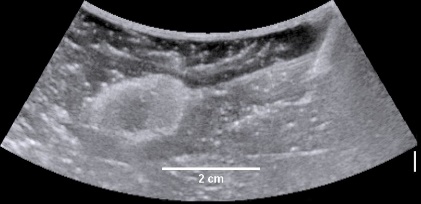** |
| --- | --- | --- |
|  | | |
| **Fig. S12.** Ultrasound images of 4mL-injected POL at 100mL/h containing 0.001 (A), 0.01 % (B), and 0.1 % (C) v/v MB. The white arrow points the needle trajectory of SEHN and the red arrow points the POL deposition in tissue. (D) Acoustic intensity, (E) acoustic heterogeneity, (F) entropy, and (G) area of injected POL with varying  % MBs. *p= 0.0389. | | |

| **Table S3.** Regression coefficient values for Multivariable linear regression on distance to needle tip and POL injection center. n=3. | |
| --- | --- |
| **Regression coefficient** | **Mean** |
| **β0 (Intercept)** | -0.5 +7.7 |
| **β1 (SEHN)** | 8.2 +7.7 |
| **β2 (MSHN)** | -5.2 +12.2 |
| **β2 (MPIN-1cm)** | 2.4 +4.0 |

| **Table S4.** One-way ANOVA of regression coefficient values from Multivariable linear regression on distance to needle tip and POL injection center. n=3. | | | | | |
| --- | --- | --- | --- | --- | --- |
| Tukey's multiple comparisons test | Mean Diff. | 95.00% CI of diff. | Below threshold? | Summary | Adjusted P Value |
| B0 vs. B1 | -8.708 | -30.76 to 13.34 | No | ns | 0.6075 |
| B0 vs. B2 | 4.708 | -17.34 to 26.76 | No | ns | 0.9005 |
| B0 vs. B3 | -2.940 | -24.99 to 19.11 | No | ns | 0.9722 |
| B1 vs. B2 | 13.42 | -8.636 to 35.47 | No | ns | 0.2822 |
| B1 vs. B3 | 5.768 | -16.28 to 27.82 | No | ns | 0.8354 |
| B2 vs. B3 | -7.648 | -29.70 to 14.40 | No | ns | 0.6936 |

| **** |
| --- |
| **Fig. S13**. Pearson correlation coefficients from the average (n=3) of the distance for needle tip to POL injection center. |

| **Table S5.** P-values of Pearson correlation coefficients from the average (n=3) of the distance for needle tip to POL injection center. | | | |
| --- | --- | --- | --- |
|  | SEHN | MSHN | MPIN-1cm |
| SEHN |  | 0.2 | 0.07 |
| MSHN | 0.2 |  | 0.04 |
| MPIN-1cm | 0.07 | 0.04 |  |

**Assessment of ultrasound imageability of POL containing DOX in *ex vivo* tissue**

|  |
| --- |
| **Fig. S14.** Solidities measured per mL of injected POL across three needle devices: SEHN, MSHN, and MPIN, |

**Transducer type evaluation of POL injected in *ex vivo* tissue**

| 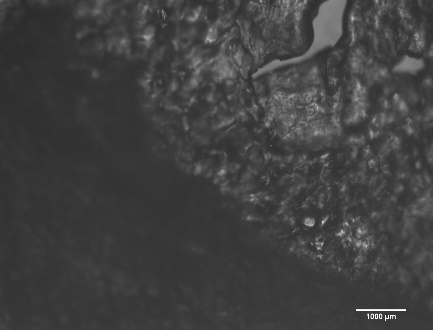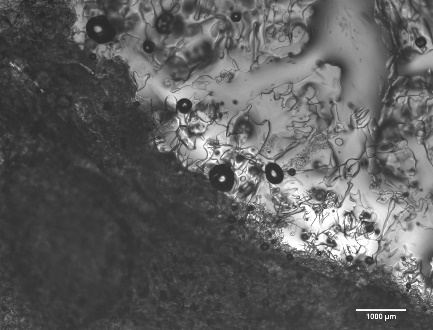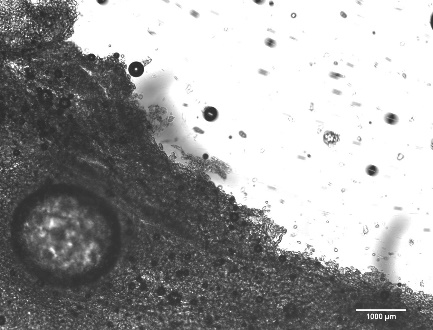  *  * |
| --- |
| **Fig. S15.** Brightfield optical image of a histological section at -20°C of *ex vivo* tissue injected with POL. The tissue section initially contained a cavity filled with gel (left). Over time at room temperature, POL transitioned to semisolid with loss of gel and finally to liquid leaving the empty cavity surrounded by tissue (right) . (*) depicts gel filling tissue cavity. |

| 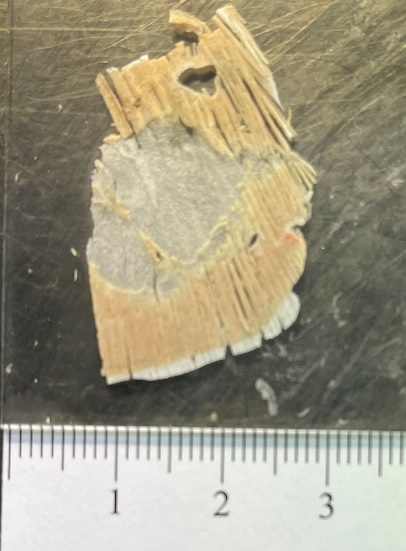  * |
| --- |
| **Fig. S16.** Gross pathology of gel injection into bovine liver and sectioned. (*) depicts gel filling tissue cavity. |

A variety of POL injections containing different concentrations of DOX (0.02,2, and 10mg/mL) (n=43 total) and without DOX (n=10 total) were performed with different imaging settings at constant 0.01% MBs content. The injections were reproducible and the results are summarized in **Table 1**. The area, circularity, and solidity across the POL injections was similar with few exceptions. For the case of the areas, statistical differences were found with 0.02mg/mL of DOX injected with MSHN (53%,55Hz) and 10mg/mL of DOX injected with SEHN (65%,55Hz) (p=0.0301). For the case of the circularities, statistical differences were found in the following conditions: POL with 10mg/mL of DOX injected with MSHN (56%,55Hz) and POL with 2mg/mL injected with SEHN (59%,55Hz) (p=0.02); POL with 0.02mg/mL DOX injected with MSHN (55%, 55Hz and 53%,55Hz) (p=0.0286); POL with 0.02mg/mL (53%,55) of DOX injected with MSHN and POL with 2mg/mL of DOX injected with SEHN (59%,55) (p=0.0015); POL with 0.02mg/ml (53%,55) of and 2mg/mL of DOX injected with SEHN (59%,55) (p=0.0015); POL with 2mg/mL of DOX (59%,55) and POL without DOX injected with SEHN (59%,55) (p=0.0338); MSHN 10mg/mL (56%,55) and SEHN 2mg/mL (59%,55) (p=0.0200); MSHN 0.02mg/mL (55%,55) and SEHN 2,g/mL (59%,55) (p=0.0007). There were not statistical differences in solidity and acoustic heterogeneity across all the tested conditions.

| **Table S6.** Imaging settings and measurements for several POL injections with and without DOX. | | | | | | | | |
| --- | --- | --- | --- | --- | --- | --- | --- | --- |
| **Device** | **DOX (mg/mL)** | **Imaging settings** | **Rate (mL/h)** | **Area (cm^2^)** | **Acoustic intensity (a.u.)** | **Circularity (a.u.)** | **Solidity (a.u)** | **n** |
| SEHN | 0 | 54%,55Hz | 100.0 | 2.8+0.5 | 123.4+27.8 | 0.8+0.1 | 0.98+0.0 | 3 |
| SEHN | 0 | 59%,55Hz | 10.0 | 3.5+0.2 | 116.4+19.8 | 0.6+0.2 | 0.89+0.1 | 3 |
| MSHN | 0 | 59%,55Hz | 10.0 | 4.2+0.9 | 105.3+23.5 | 0.7+0.1 | 0.94+0.0 | 4 |
| MPIN-1cm | 0.02 | 49%,55Hz | 10.0 | 3.4+0.1 | 121.8+7.1 | 0.8+0.1 | 0.95+0.0 | 3 |
| SEHN | 0.02 | 56%,56Hz | 10.0 | 3.9+0.5 | 73.0+27.2 | 0.8+0.0 | 0.97+0.0 | 3 |
| MSHN | 0.02 | 56%,55Hz | 10.0 | 2.8+0.9 | 70.2+23.8 | 0.7+0.1 | 0.94+0.0 | 3 |
| SEHN | 0.02 | 53%,55Hz | 1000.0 | 3.2+0.1 | 124.7+14.6 | 0.6+0.1 | 0.94+0.0 | 8 |
| MSHN | 0.02 | 55%,55Hz | 1000.0 | 2.8+0.8 | 109.5+24.3 | 0.5+0.1 | 0.93+0.0 | 3 |
| MSHN | 0.02 | 53%,55Hz | 1000.0 | 2.5+0.5 | 86.8+19.3 | 0.8+0.4 | 0.96+0.1 | 4 |
| SEHN | 2 | 59%55Hz | 1000.0 | 3.5+0.4 | 81.6+8.6 | 0.8+0.0 | 0.97+0.0 | 3 |
| SEHN | 2 | 60%,55Hz | 1000.0 | 3.1+0.9 | 83.2+11.6 | 0.8+0.0 | 0.96+0.0 | 3 |
| SEHN | 10 | 40%,46Hz | 100.0 | 3.8+0.7 | 147.6+4.6 | 0.7+0.1 | 0.94+0.0 | 3 |
| SEHN | 10 | 65%,55Hz | 10.0 | 4.6+0.4 | 176.3+4.3 | 0.7+0.2 | 0.90+0.1 | 4 |
| MSHN | 10 | 56%,55HZ | 10.0 | 3.2+0.8 | 117.1+38.5 | 0.7+0.1 | 0.92+0.0 | 6 |

**Experimental Section**

| 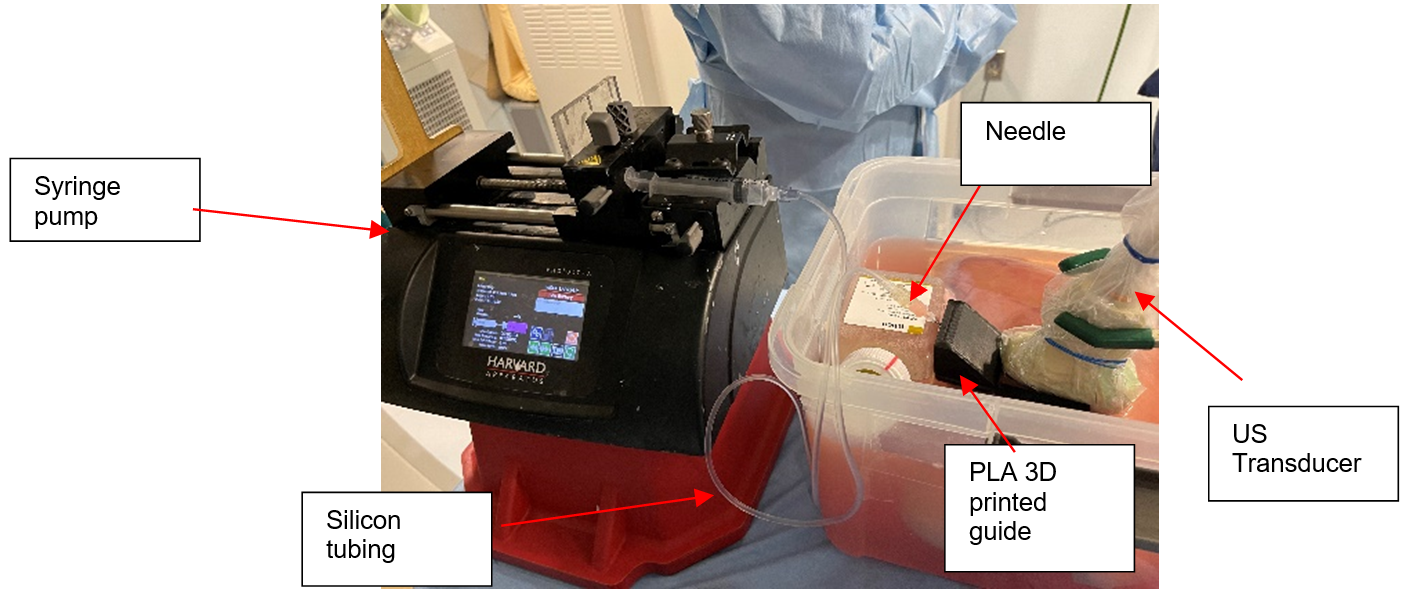 |
| --- |
| **Fig. S17.** Ex vivo liver model injection setup |
